# The mechanism of lineage-specific tRNA recognition by bacterial tryptophanyl-tRNA synthetase and its implications for inhibitor discovery

**DOI:** 10.1093/nar/gkaf466

**Published:** 2025-06-04

**Authors:** Xiaoying Peng, Kaijiang Xia, Lingzhen Xiao, Haoran Qi, Qingting Huang, Manli Xiang, Lu Han, Haipeng Qiu, Qiong Gu, Bingyi Chen, Huihao Zhou

**Affiliations:** State Key Laboratory of Anti-Infective Drug Discovery and Development, School of Pharmaceutical Sciences, Sun Yat-sen University, Guangzhou 510006, China; Guangdong Provincial Key Laboratory of Chiral Molecule and Drug Discovery, School of Pharmaceutical Sciences, Sun Yat-sen University, Guangzhou 510006, China; State Key Laboratory of Anti-Infective Drug Discovery and Development, School of Pharmaceutical Sciences, Sun Yat-sen University, Guangzhou 510006, China; Guangdong Provincial Key Laboratory of Chiral Molecule and Drug Discovery, School of Pharmaceutical Sciences, Sun Yat-sen University, Guangzhou 510006, China; State Key Laboratory of Anti-Infective Drug Discovery and Development, School of Pharmaceutical Sciences, Sun Yat-sen University, Guangzhou 510006, China; Guangdong Provincial Key Laboratory of Chiral Molecule and Drug Discovery, School of Pharmaceutical Sciences, Sun Yat-sen University, Guangzhou 510006, China; State Key Laboratory of Anti-Infective Drug Discovery and Development, School of Pharmaceutical Sciences, Sun Yat-sen University, Guangzhou 510006, China; Guangdong Provincial Key Laboratory of Chiral Molecule and Drug Discovery, School of Pharmaceutical Sciences, Sun Yat-sen University, Guangzhou 510006, China; State Key Laboratory of Anti-Infective Drug Discovery and Development, School of Pharmaceutical Sciences, Sun Yat-sen University, Guangzhou 510006, China; Guangdong Provincial Key Laboratory of Chiral Molecule and Drug Discovery, School of Pharmaceutical Sciences, Sun Yat-sen University, Guangzhou 510006, China; State Key Laboratory of Anti-Infective Drug Discovery and Development, School of Pharmaceutical Sciences, Sun Yat-sen University, Guangzhou 510006, China; Guangdong Provincial Key Laboratory of Chiral Molecule and Drug Discovery, School of Pharmaceutical Sciences, Sun Yat-sen University, Guangzhou 510006, China; State Key Laboratory of Anti-Infective Drug Discovery and Development, School of Pharmaceutical Sciences, Sun Yat-sen University, Guangzhou 510006, China; Guangdong Provincial Key Laboratory of Chiral Molecule and Drug Discovery, School of Pharmaceutical Sciences, Sun Yat-sen University, Guangzhou 510006, China; State Key Laboratory of Anti-Infective Drug Discovery and Development, School of Pharmaceutical Sciences, Sun Yat-sen University, Guangzhou 510006, China; Guangdong Provincial Key Laboratory of Chiral Molecule and Drug Discovery, School of Pharmaceutical Sciences, Sun Yat-sen University, Guangzhou 510006, China; State Key Laboratory of Anti-Infective Drug Discovery and Development, School of Pharmaceutical Sciences, Sun Yat-sen University, Guangzhou 510006, China; State Key Laboratory of Anti-Infective Drug Discovery and Development, School of Pharmaceutical Sciences, Sun Yat-sen University, Guangzhou 510006, China; Guangdong Provincial Key Laboratory of Chiral Molecule and Drug Discovery, School of Pharmaceutical Sciences, Sun Yat-sen University, Guangzhou 510006, China; State Key Laboratory of Anti-Infective Drug Discovery and Development, School of Pharmaceutical Sciences, Sun Yat-sen University, Guangzhou 510006, China; Guangdong Provincial Key Laboratory of Chiral Molecule and Drug Discovery, School of Pharmaceutical Sciences, Sun Yat-sen University, Guangzhou 510006, China

## Abstract

Tryptophanyl-tRNA synthetase (TrpRS) catalyzes the attachment of tryptophan (l-Trp) to tRNA^Trp^, thereby providing the ribosome with a crucial substrate for the decoding of the UGG codon during protein translation. Both bacterial and eukaryotic TrpRSs are unable to efficiently cross-aminoacylate their respective tRNA^Trp^ substrates, indicating the evolution of lineage-specific mechanisms for tRNA^Trp^ recognition. Herein, we present the first co-crystal structure of bacterial TrpRS from *Escherichia coli* (*Ec*TrpRS) in complex with its tRNA^Trp^. *Ec*TrpRS demonstrates bacterial-specific interactions with both the anticodon triplet and the acceptor arm of tRNA^Trp^. Particularly, the bacterial-specific residue Glu155 forms hydrogen bonds with the discriminator base G73, thereby stabilizing it in a conformation distinct from that of A73 in the eukaryotic tRNA^Trp^ bound to human TrpRS. Through compound screening, we identified tirabrutinib and its analogues as selective inhibitors of bacterial TrpRS. These compounds occupy the l-Trp and tRNA^Trp^ CCA end binding sites of bacterial TrpRS, both of which exhibit less conservation compared to the ATP binding site between bacterial and eukaryotic TrpRSs. These findings enhance our understanding of the lineage-specific recognition of tRNA^Trp^ by bacterial TrpRS and highlight the CCA end binding site as a promising target for the future development of selective bacterial TrpRS inhibitors as potential antimicrobials.

## Graphical Abstract

**Graphical Abstract:**
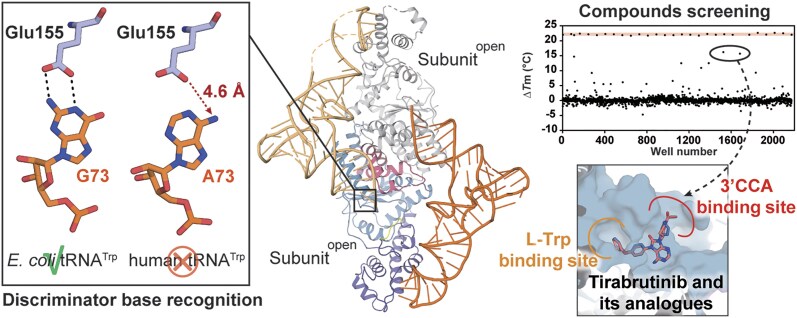

## Introduction

The accurate ribosomal translation of genetic information encoded in messenger RNA transcripts into amino acid sequences of nascent peptides relies on the precise attachment of proteinogenic amino acids to their cognate transfer RNAs (tRNAs) [1]. This reaction is catalyzed by a family of ancient enzymes known as aminoacyl-tRNA synthetases (AARSs) [2, 3]. Among these, tryptophanyl-tRNA synthetase (TrpRS) facilitates to decode the UGG codon by linking tryptophan (l-Trp) to tRNA^Trp^, thereby playing an essential role in the protein translation process across all forms of cellular life [4].

Substantial studies have been conducted on TrpRS to elucidate the catalytic mechanism underlying the tryptophanylation reaction [5–11]. This reaction comprises two steps: first, the activation of l-Trp with ATP, resulting in the formation of the intermediate product tryptophanyl adenylate (Trp-AMP); second, the transfer of the l-Trp moiety to the 3′ end nucleotide A76 of tRNA^Trp^, yielding Trp-tRNA^Trp^ [12]. While most class I AARS members are monomers, TrpRS functions as a homodimer [10]. Similar to many other dimeric AARSs, TrpRS exhibits half-of-the-sites reactivity [13–16]. Our recent study has captured an “open–closed” asymmetric structure of *Escherichia coli* TrpRS (*Ec*TrpRS), wherein the closed subunit binds Trp-AMP while the open subunit remains empty (PDB ID 8I1W), supporting the half-of-the-sites reactivity of bacterial TrpRS in the first step of the catalytic process [17]. Notably, this asymmetric conformation may be attributed to the idea that at least one of the two subunits in bacterial TrpRS must adopt an open conformation to enable the functional binding of tRNA^Trp^, thereby initiating the second step of the catalytic reaction [17]. However, this hypothesis regarding bacterial TrpRS is in conflict with the previous observations in the tRNA^Trp^-bound human cytoplasmic TrpRS (*Hc*TrpRS) that adopts a “closed–closed” conformation, and currently lacks structural evidence.

**Figure 1.**
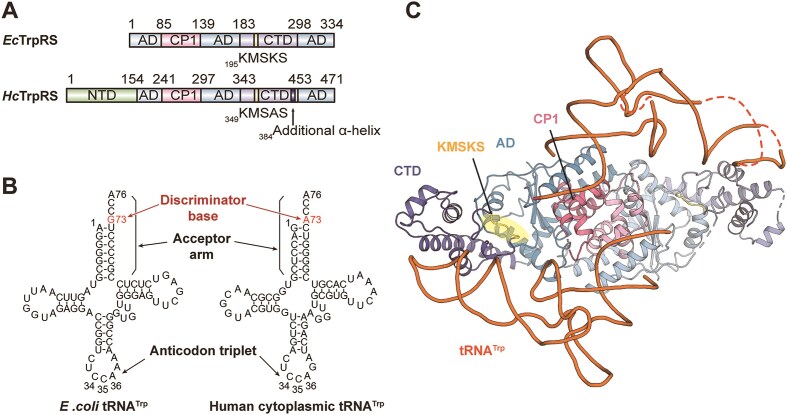
The structure of the *Ec*TrpRS·tRNA^Trp^ complex. (**A**) The domain organization of *Ec*TrpRS and *Hc*TrpRS. (**B**) Cloverleaf models of *E. coli* tRNA^Trp^ and human cytoplasmic tRNA^Trp^. (**C**) The cartoon presentation of the homodimeric *Ec*TrpRS in complex with two *E. coli* tRNA^Trp^ molecules reveals a symmetric cross-subunit binding mode.

Notably, bacterial and eukaryotic TrpRSs display considerable sequence variations in both their overall domain organization and key residues in active sites (Fig. 1A), resulting in divergent substrate recognition mechanisms. For example, the indole nitrogen of the substrate l-Trp is recognized through hydrogen bonding (H-bonding) with an aspartate residue in an α-helix when binding to bacterial TrpRS, whereas it interacts with a tyrosine residue in a β-strand when binding to eukaryotic TrpRS [18]. Importantly, bacterial tRNA^Trp^ has a guanine at the position 73 (G73), while eukaryotic tRNA^Trp^ possesses A73 (Fig. 1B). This position serves as a key discriminator between the two tRNA^Trp^ molecules, and bacterial and eukaryotic TrpRSs cannot efficiently cross-aminoacylate their tRNA^Trp^ substrates [19]. It was observed that human TrpRS charges the non-cognate *Bacillus subtilis* tRNA^Trp^ with an efficiency 91-fold lower than that of human tRNA^Trp^, while conversely, human tRNA^Trp^ is catalyzed by *B*.*subtilis* TrpRS (*Bs*TrpRS) 132-fold less efficiently compared to *B. subtilis* tRNA^Trp^ [19]. The co-crystal structure of *Hc*TrpRS in complex with bovine tRNA^Trp^ has revealed that the N-terminal domain (NTD) is responsible for recognizing A73 in eukaryotic tRNA^Trp^ [20]. In contrast, bacterial TrpRS lacks the NTD (Fig. 1A). To date, the high-resolution structural model regarding the lineage-specific recognition of bacterial tRNA^Trp^ by bacterial TrpRS is still lacking.

Owing to their fundamental biological roles and considerable structural differences from their human counterparts, bacterial AARSs have long been recognized as promising drug targets for combating microbial infections [21–24]. For instance, mupirocin, an inhibitor of bacterial isoleucyl-tRNA synthetase (IleRS), is widely utilized in the treatment of skin infections caused by Gram-positive bacteria [25, 26]. For bacterial TrpRS, two natural products, indolmycin and chuangxinmycin, have demonstrated potent inhibitory activity, while showing minimal interactions with *Hc*TrpRS [27, 28]. However, the clinical application of these compounds has been hindered by issues related to insufficient permeability and a limited antibacterial spectrum [29, 30]. Notably, both compounds primarily occupy the l-Trp binding site of bacterial TrpRS. The potential differences in the mechanisms of tRNA^Trp^ recognition between bacterial and eukaryotic TrpRSs may offer additional opportunities for the discovery of bacterial-specific TrpRS inhibitors as potential antimicrobial agents.

This study reports the first co-crystal structure of bacterial TrpRS in complex with tRNA^Trp^. Although an “open–closed” asymmetric dimer of *Ec*TrpRS was utilized for crystallization, the resulting structure of *Ec*TrpRS adopts an “open–open” conformation to symmetrically capture two tRNA^Trp^ molecules through a cross-subunit binding mode. *Ec*TrpRS employs several bacterial-specific interactions to recognize both the anticodon triplet and the acceptor arm of tRNA^Trp^, particularly the discriminator nucleotide G73. Furthermore, a novel class of bacterial-selective TrpRS inhibitors has been identified, and structural and biophysical analyses revealed that they may simultaneously compete with both l-Trp and the CCA end of tRNA^Trp^.

## Materials and methods

### Protein preparation

The DNA sequence encoding the full-length *Ec*TrpRS (UniProt ID P00954) was amplified from the genomic DNA of the *E*.*coli* K12 strain and cloned into the pET20b(+) plasmid (Novagen) with a hexahistidine tag at the C-terminus. *Escherichia coli* BL21(DE3) cells transformed with the *Ec*TrpRS-pET20b(+) plasmid were cultured in Luria–Bertani medium supplemented with 100 mg/l ampicillin at 37°C until OD_600_ of 0.6–0.8 was reached. 0.15 mM isopropyl-β-d-thiogalactoside (IPTG) was added to induce the overexpression of *Ec*TrpRS at 16°C for 12 h. The *E*.*coli* cells were harvested by centrifugation, resuspended in a washing buffer [400 mM NaCl, 50 mM Tris–HCl (pH 8.0), 5% glycerol, and 30 mM imidazole], and lysed through sonication. The resulting cell lysate was centrifuged at 18 000 rpm for 40 min, and the supernatant was transferred to the Ni-NTA column pre-equilibrated with the washing buffer. Impurities were removed by washing with 20 column volumes of the washing buffer. The target protein was then eluted using 5 column volumes of an elution buffer [400 mM NaCl, 50 mM Tris–HCl (pH 8.0), 5% glycerol, and 200 mM imidazole]. The target protein was concentrated and further purified using HiLoad 16/60 Superdex 200 pg column (GE Healthcare) with a running buffer [200 mM NaCl, 20 mM Tris–HCl (pH 8.0), 5% glycerol, 1 mM dithiothreitol (DTT), and 1 mM ethylenediaminetetraacetic acid (EDTA)]. Protein purity was verified using sodium dodecyl sulfate–polyacrylamide gel electrophoresis (SDS–PAGE). The target protein was concentrated to 60 mg/ml in a storage buffer [50 mM NaCl, 2 mM Tris–HCl (pH 8.0), 5% glycerol] and stored at −80°C. *Ec*TrpRS variants and *Hc*TrpRS (UniProt ID P23381) were expressed and purified similarly to *Ec*TrpRS.

### *In vitro* transcription and purification of tRNA

The DNA templates for tRNA^Trp^ transcription were generated through an initial polymerase chain reaction (PCR) using Primer1-1 (5′-**TAATACGACTCACTATA**AGGGGCGTAGTTCAATTGGTAGAGCACCGGTCTCCAAAACC-3′) and Primer1-2 (5′-TGGCAGGGGCGGAGAGACTCGAACTCCCAACACCCGGTTTTGGAGACCGGTGCT-3′). These two primers encompass the full length of the *E*.*coli* tRNA^Trp^ gene and exhibit partial complementarity to one another (underlined nucleotides), with Primer1-1 containing a T7 promoter (nucleotides in bold). The PCR products were subsequently amplified in a second round of PCR using Primer1-3 (5′-TAATACGACTCACTATAAGGGGCGTAG-3′) and Primer1-4 (5′-*UG*GCAGGGGCGGAGAGACTCGA-3′). Notably, the 2′ hydroxyl groups of the first two nucleotides (nucleotides in italics) at the 5′ end of Primer4 are methylated to prevent the incorporation of additional nucleotides by T7 RNA polymerase [31]. The preparation methods for the DNA templates of tRNA^Trp^(ΔA76), tRNA^Trp^(ΔC75A76), and tRNA^Trp^(ΔC74C75A76) are similar. All the primers are listed in Supplementary Table S3.

The product of the second round of PCR, without additional purification, was used as the DNA template for the *in vitro* transcription of tRNA^Trp^. A transcription reaction of 10 ml was prepared by mixing 2 ml of the PCR product, 2 ml of a 5× transcription buffer [1 M Tris–HCl (pH 8.0), 10 mM spermidine, and 50 mM DTT], 1 ml of NTPs (40 mM each), 0.2 ml of 1 M MgCl_2_, 0.2 ml of 10 mg/ml T7 polymerase, and 3.6 ml of DEPC-treated water. The reaction was incubated at 37°C for 4 h. The transcripts were mixed with 500 μl of 0.5 M EDTA, and placed at room temperature until clarified. The mixture was then mixed with two volumes of anhydrous ethanol, and incubated at −20°C overnight to facilitate the precipitation of the tRNA products. The precipitate was collected via centrifugation and subsequently dissolved in 2 ml DEPC-treated water. An additional 2 ml urea–PAGE loading buffer (95% formamide, 20 mM EDTA) was added, and the transcript was then purified using a 12% polyacrylamide gel supplemented with 8 M urea. The tRNA^Trp^ band observed was cut under the UV lamp, and was extracted with 0.5 M ammonium acetate and precipitated with ethanol at −20°C overnight. The tRNA^Trp^ precipitates were collected through centrifugation and dissolved in a buffer containing 20 mM Tris (pH 8.0) and 1 mM EDTA. The dissolved tRNA was heated at 65°C for 5 min, and then gradually cooled to room temperature after the addition of 10 mM MgCl_2_ for refolding. The refolded tRNA^Trp^ was concentrated to 10 mg/ml using a 10-kDa Ultra-4 centrifugal filtration unit (Millipore) and subsequently stored at −80°C for future applications.

### Crystallography

The crystals were grown using the sitting-drop vapor-diffusion method. Prior to setting up the crystallization drops, *Ec*TrpRS (15 mg/ml) was preincubated with tRNA^Trp^ (7.5 mg/ml) on ice for 30 min to form the *Ec*TrpRS·tRNA^Trp^ complex. Each sitting drop comprised 1 μl of *Ec*TrprS·tRNA^Trp^ complex and 1 μl of the reservoir solution [0.5 M ammonium acetate, 0.1 M HEPES (pH 7.5), 16% (w/v) PEG 3350], and was equilibrated against 100 μl of reservoir solution at 18°C for 5–7 days. Microseeding was employed to grow large crystals for data collection. For the co-crystallization of *Ec*TrpRS in complex with various compounds, *Ec*TrpRS (25 mg/ml) was preincubated with 1 mM of the target compound on ice for 30 min, followed by crystallization using the reservoir solution containing 0.15 M ammonium sulfate, 0.1 M HEPES (pH 7.5), and 25% (w/v) PEG 3350. Prior to data collection, the crystals were rapidly immersed in a cryoprotectant solution (the reservoir solution supplemented with 20% ethylene glycol) for a few seconds and subsequently flash-frozen in liquid nitrogen.

Diffraction data were collected at 100 K with a wavelength of 0.979 Å at the BL19U1 beamline of the National Facility for Protein Sciences Shanghai (NFPSS), the Shanghai Synchrotron Radiation Facility (SSRF). The data were processed using XDS [32] and Aimless [33]. The structures were solved through molecular replacement, utilizing the *Ec*TrpRS structure (PDB ID 8I4I) as the search model within the Molrep program [34]. Iterative refinement of the structural models was conducted using Coot [35] and Refmac5 [36]. The stereochemical quality of the final models was assessed using MolProbity [37]. The statistics for data collection and structural refinement are listed in Supplementary Table S1.

### ATP consumption assay

The aminoacylation activities of *Ec*TrpRS and its variants to tRNA^Trp^ were evaluated using an ATP consumption assay [38]. In brief, the 100 μl reaction contained 50 nM *Ec*TrpRS, 4 μM ATP, 50 μM l-Trp, 0.2 g/l tRNA^Trp^ (prepared by *in vitro* transcription), 30 mM HEPES (pH 7.5), 150 mM NaCl, 30 mM KCl, 40 mM MgCl_2_, 1 mM DTT, and 0.1% bovine serum albumin, and was incubated at 37°C. At various time points (2, 5, 10, 20, and 30 min), 5 μl aliquots were transferred to a 384-well plate and combined with 5 μl of Kinase-Glo^®^ Max Reagent (Promega). After 10 min of incubation, luminescence (*L*) was measured using a Synergy H1 microplate reader (BioTek). The reaction without the addition of tRNA^Trp^ served as a control (*L*_c_). ATP consumption (μM) = 4 × (1 − *L*/*L*_c_). Each reaction was conducted in triplicate, and the results are expressed as means ± standard deviations (SDs) (*n* = 3). Statistical analyses were performed using GraphPad Prism 9 software, and a one-phase association model was utilized to fit the time response curves (ATP consumption versus reaction time).

### Fluorescence-based thermal shift assay

The melting temperature (*T*_m_) represents the thermal stability of a protein and can be measured using a fluorescence dye to detect the exposure of hydrophobic residues during the protein denaturation process. Ligand binding typically slows the denaturation of a protein by providing additional stabilization energy, and the change in protein’s *T*_m_, denoted as Δ*T*_m_, exhibits a positive correlation with the affinity of the ligand to the protein [39, 40]. Thus, a fluorescence-based thermal shift assay (TSA) was employed to screen molecules against *Ec*TrpRS. The reaction solutions, which comprised 2 μg *Ec*TrpRS, 4× SYPRO Orange fluorescence dye (Sigma–Aldrich), and 100 μM of the tested compounds [in 1% dimethyl sulfoxide (DMSO)] in TSA buffer [150 mM NaCl, 100 mM MES (pH 6.5)], were prepared in 96-well plates (Life Technologies) with a total volume of 20 μl. The plates were gradually heated from 25°C to 95°C at a rate of 1°C/min. Fluorescence intensity was recorded every 20 s using StepOne Plus™ RT-PCR equipment (Life Technologies). The *T*_m_ values were calculated using StepOne™ software v2.3 by averaging the results from triplicate assays. Indolmycin served as a positive control, while 1% DMSO was used as a negative control.

### Pyrophosphate production assay

The inhibitory effects of various compounds of *Ec*TrpRS were evaluated using a pyrophosphate (PPi) production assay [41]. *Ec*TrpRS was incubated with 10 μM (in 0.1% DMSO) of each compound for 10 min at room temperature. Each reaction had a total volume of 160 μl and consisted of 30 nM *Ec*TrpRS, 40 μM ATP, 3 μM l-Trp, 100 mM NH_2_OH, and 0.05 mg/ml pyrophosphatase in the reaction buffer [30 mM HEPES (pH 7.5), 150 mM NaCl, 40 mM MgCl_2_, and 1 mM DTT]. The mixture was incubated at 26°C for 45 min, after which 40 μl of malachite green reagent was added to terminate the reaction and develop color. Following an additional incubation of 10 min, the absorbance (*A*) was measured at 640 nm using a Synergy H1 microplate reader (BioTek). The absorbance of the reaction without adding a compound was designated as *A*_1_, the absorbance of the reaction without adding *Ec*TrpRS was *A*_0_, and the absorbance of the reaction with the addition of a compound was *A*_C_. The inhibitory rates of compounds on enzyme activity were calculated as (*A*_1_ − *A*_C_)/(*A*_1_ − *A*_0_) × 100%. Each reaction was performed in triplicate.

### Isothermal titration calorimetry assay

The binding affinities of various compounds to *Ec*TprRS were measured using a MicroCal VP-ITC microcalorimeter (MicroCal). Compounds at a concentration of 200 μM were titrated into 20 μM of *Ec*TrpRS in PBS (phosphate buffered saline) buffer (137 mM NaCl, 2.7 mM KCl, 8 mM Na_2_HPO_4_, and 2 mM KH_2_PO_4_). The titration assays were conducted at 26°C, beginning with an initial injection of 0.4 μl, followed by 18 injections of 2 μl each, with an interval of 120 s between each injection. The dissociation constants (*K*_d_) were calculated by fitting the calorimetric data to a one-site binding model using MicroCal PEAQ-ITC analysis software, with the reported errors in *K*_d_ values representing the curve fitting errors. All isothermal titration calorimetry (ITC) experiments were repeated at least twice, and the titrations of compounds to *Hc*TrpRS were also performed for comparison.

## Results

### The cross-subunit capturing of two tRNA^Trp^ molecules by *Ec*TrpRS dimer

The full-length *Ec*TrpRS in complex with *E*.*coli* tRNA^Trp^ was successfully crystallized, and the structure was resolved to a resolution of 2.8 Å, with the *R*_work_ and *R*_free_ factors of 0.236 and 0.271, respectively (Fig. 1C and Supplementary Table S1). The asymmetric unit contains a functional homodimer of *Ec*TrpRS bound with two molecules of tRNA^Trp^ via a cross-subunit binding mode, whereas the anticodon loop of one tRNA^Trp^ molecule interacts with one *Ec*TrpRS subunit and its acceptor arm binds to the other subunit.

Each *Ec*TrpRS subunit comprises an aminoacylation domain (AD; residues 1–85, 140–183, and 299–334), a connecting polypeptide 1 domain (CP1; residues 86–139), and a C-terminal α-helical domain (CTD; residues 184–298) (Fig. 1A and C). The AD adopts a classical Rossmann fold and interacts with the acceptor arm of *E*.*coli* tRNA^Trp^. The insertion sequence CP1 within the AD is critical for establishing dimeric interfaces between the two *Ec*TrpRS subunits, and it also makes significant contributions to the formation of binding pocket of substrate l-Trp [17] as well as the recognition of the acceptor arm of tRNA^Trp^. The CTD, characterized by an α-helical bundle, interacts with the anticodon triplet of tRNA^Trp^ (Fig. 1C). The structure and the interactions with tRNA^Trp^ are nearly identical between two subunits of *Ec*TrpRS, with a root-mean-square deviation (RMSD) of 0.603 Å for 275 comparable Cα atoms, making the *Ec*TrpRS·tRNA^Trp^ complex adopt an overall symmetrical conformation (Supplementary Fig. S1A).

In previous research, the tRNA^Trp^-free *Ec*TrpRS has been crystallized in both symmetric “open–open” (PDB ID 5V0I) and asymmetric “open–closed” (PDB ID 8I1W) conformations [17]. Both active site cavities of the “open–open” *Ec*TrpRS bind l-Trp and AMP; conversely, the active site within the closed subunit of the “open–closed” *Ec*TrpRS is occupied by an intermediate product, whereas the other active site within the open subunit remains empty. Structural superimposition revealed that the tRNA^Trp^-bound *Ec*TrpRS adopts an “open–open” conformation, exhibiting an overall RMSD of 0.527 Å for 529 comparable Cα atoms when compared to the “open–open” tRNA^Trp^-free *Ec*TrpRS (Supplementary Fig. S1B). The tRNA^Trp^-bound “open–open” conformation of *Ec*TrpRS is distinct from the “closed–closed” conformation observed for tRNA^Trp^-bound eukaryotic *Hc*TrpRS (PDB ID 2DR2) [20], and supports our previous hypothesis that bacterial TrpRS requires at least one subunit to adopt an open conformation to functionally interact with tRNA^Trp^ [17].

### The recognition of the anticodon triplet by *Ec*TrpRS

**Figure 2.**
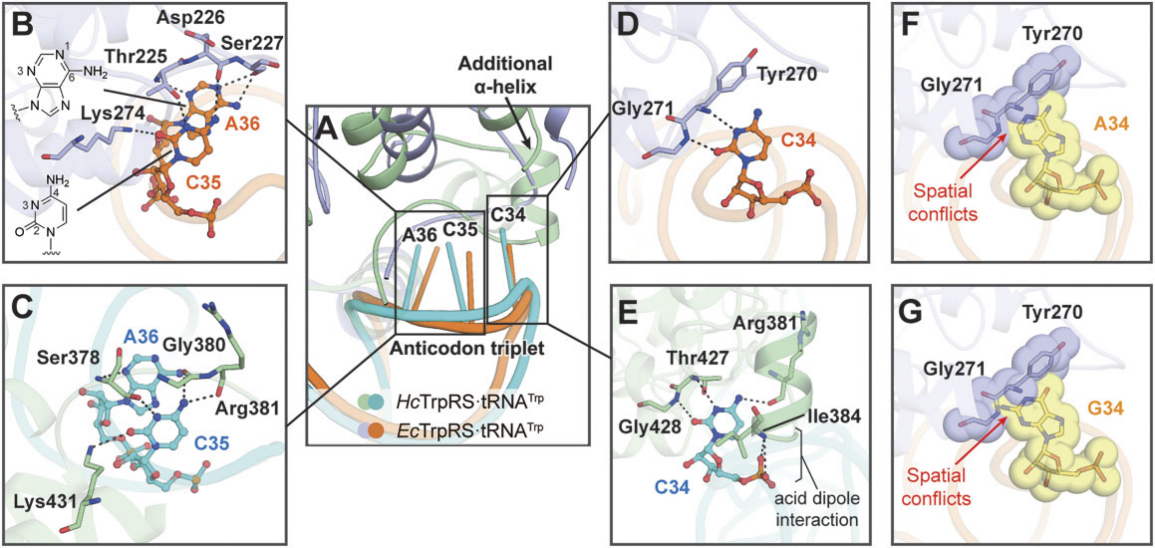
The recognition of the anticodon triplet of tRNA^Trp^ by *Ec*TrpRS. (**A**) The *Ec*TrpRS·tRNA^Trp^ complex displays structural variations compared to the *Hc*TrpRS·tRNA^Trp^ complex (PDB ID 2DR2) at their anticodon binding sites. The recognition of C35 and A36 by *Ec*TrpRS (**B**) and *Hc*TrpRS (**C**). The recognition of C34 by *Ec*TrpRS (**D**) and *Hc*TrpRS (**E**). *In silico* mutation of C34 to A34 (**F**) or G34 (**G**) created spatial conflicts with the residues Tyr270 and Gly271 of *Ec*TrpRS. The nucleotides and amino acids are shown as stick models with transparent spheres.

The anticodon triplet serves as the identity element of tRNA^Trp^ during its recognition by TrpRS [42, 43]. In the co-crystal structure of the *Ec*TrpRS·tRNA^Trp^ complex, the anticodon triplet of *E*.*coli* tRNA^Trp^ is primarily recognized by a loop (comprising residues Arg222 to Ser227) within the CTD of *Ec*TrpRS (Fig. 2A). Thr225, Asp226, and Ser227 in this loop, together with Lys274, establish an extensive hydrogen bonding network to facilitate the recognition of C35 and A36. Specifically, the O2 (H-bond acceptor), N3 (H-bond acceptor), and N4 (H-bond donor) atoms of C35 are recognized through H-bonding with Lys274, Thr225, and Asp226, respectively; the N1 (H-bond acceptor), N3 (H-bond acceptor), N6 (H-bond donor) atoms of A36 form three H-bonds with the hydroxyl group of Ser227 and the main chain nitrogen of Thr225 (Fig. 2B). The residues Thr225, Asp226, Ser227, and Lys274 are conserved among bacterial TrpRS, as revealed by multiple sequence alignments (Supplementary Fig. S2). Correspondingly, *Hc*TrpRS specifically recognizes C35 and C36 using Ser378, Gly380, Arg381, and Lys431 (corresponding to Thr225, Ser227, Asp228, and Lys274 of *Ec*TrpRS, respectively), with the majority of H-bonding interactions targeting C35 and only a single H-bond recognizing A36 (Fig. 2C) [20]. Notably, in comparison to Gly380 of *Hc*TrpRS, the hydroxyl group of the corresponding Ser227 in *Ec*TrpRS contributes two H-bonds to recognize the two Watson–Crick pairing sites (N1 and N6 atoms) of A36, making A36 play a more important role in the recognition of the anticodon triplet of tRNA^Trp^ by bacterial TrpRS than by eukaryotic TrpRS.

In the *Ec*TrpRS·tRNA^Trp^ complex, the O2 and N3 atoms of C34 are recognized through two H-bonds with the backbones of Gly271 and Tyr270, respectively (Fig. 2D). In contrast, in the *Hc*TrpRS·tRNA^Trp^ complex, in addition to the two corresponding H-bonds with Gly428 and Thr427 (corresponding to Gly271 and Tyr270 of *Ec*TrpRS, respectively), Arg381 contributes an additional H-bond to recognize the N4 atom of C34 (Fig. 2E). Moreover, compared to *Ec*TrpRS, the CTD of *Hc*TrpRS contains an additional eukaryotic-specific short α-helix (residues 384–391) [19], which contributes to stabilizing C34 by forming an acid–α-helix dipole interaction with its backbone phosphate (PDB ID 2DR2) (Fig. 2E). The absence of this additional α-helix in *Ec*TrpRS results in less interactions with the “wobble” nucleotide C34. However, it is important to note that although *Ec*TrpRS does not bind C34 as tightly as *Hc*TrpRS, it still ensures the reliable identification of C34, primarily through size discrimination. When C34 was mutated to guanine and adenine *in silico*, both purines exhibited significant spatial conflicts with Tyr270 and Gly271 (Fig. 2F and G), avoiding the mischarge of tRNA^Cys^. Additionally, it is noteworthy that *Ec*TrpRS does not require discrimination between C34 and U34, as UGA serves as a stop codon.

### The recognition of the discriminator base G73 by Glu155

While the anticodon triplet functions as the identity element of tRNA^Trp^ in all cellular life, the nucleotide 73 located between the acceptor stem and the 3′ CCA end has been identified as the major discriminator that ensures lineage-specific recognition and charging of tRNA^Trp^ [19, 44]. Additionally, the sequences of the acceptor stem in tRNA^Trp^ molecules from bacteria and eukaryotes exhibit significant differences and serve as minor discriminators for tRNA^Trp^ from different domains of life [19, 45].

**Figure 3.**
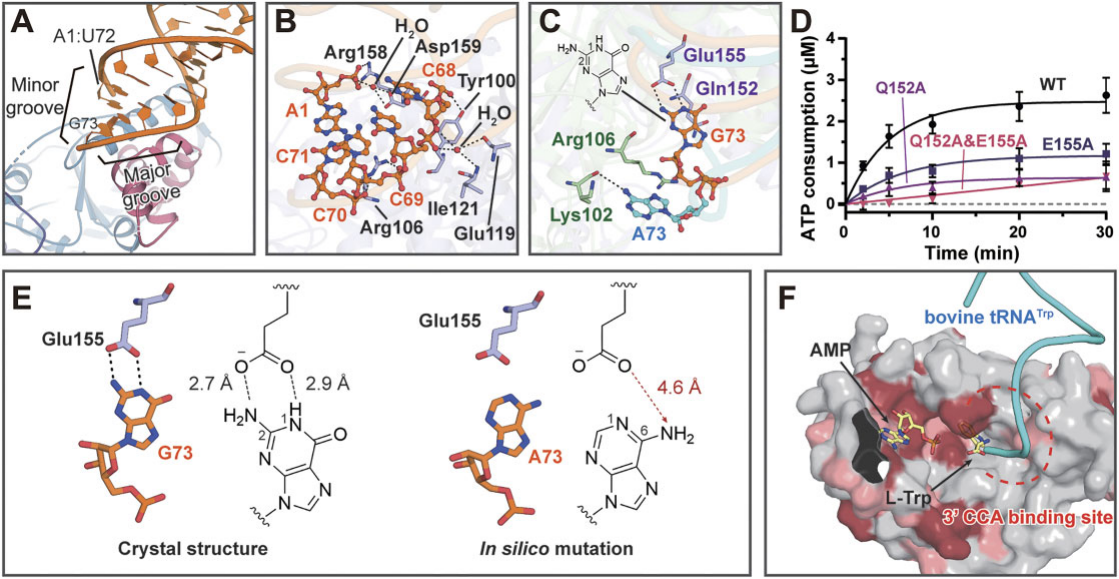
Recognition of the tRNA^Trp^ acceptor arm by *Ec*TrpRS. (**A**) *Ec*TrpRS interacts with the acceptor stem of tRNA^Trp^ from the major groove. (**B**) The charge–charge interactions between *Ec*TrpRS and the acceptor stem of tRNA^Trp^. Notably, no base-specific interactions were observed for the minor identity elements A1:U72 and G5:C68 within the acceptor stem. (**C**) Structural comparison revealed distinct orientations for the major discriminator base G73 of *E*. *coli* tRNA^Trp^ in the *Ec*TrpRS·tRNA^Trp^ complex (protein in light purple and tRNA^Trp^ in orange) and A73 of bovine tRNA^Trp^ in the *Hc*TrpRS·tRNA^Trp^ complex (PDB ID 2DR2) (protein in green and tRNA^Trp^ in cyan). (**D**) The aminoacylation activity of *Ec*TrpRS and its variants. The mutations Q152A, Q155A, and Q152A&E155A resulted in a significant reduction in the charging activity of *Ec*TrpRS for *E*.*coli* tRNA^Trp^. (**E**) The discriminator G73 of *E*.*coli* tRNA^Trp^ is recognized by Glu155 of *Ec*TrpRS through two H-bonds. *In silico* mutation of G73 to A73 results in the loss of H-bonding interactions with Glu155. (**F**) The 3′ CCA end of bovine tRNA^Trp^ (cyan) was modeled into the active site cavity of *Ec*TrpRS based on the structure of the *Hc*TrpRS·tRNA^Trp^ complex. The surface of the active site cavity of *Ec*TrpRS is drawn, with conserved residues between *Ec*TrpRS and *Hc*TrpRS being colored in dark red, similar residues in light red, and non-conserved residues in gray.

In the co-crystal structure of the *Ec*TrpRS·tRNA^Trp^ complex, *Ec*TrpRS interacts with first five base pairs of the acceptor stem of tRNA^Trp^ from the major groove, primarily using two α-helices located within the AD and CP1 domains (Fig. 3A). Rather than exhibiting base specificity, these interactions predominantly involve the phosphate backbone of the nucleotides. Specifically, the phosphates of A1, C68, C70, and C71 are directly recognized by Arg158, Tyr100, and Arg106, respectively, through ionic and H-bonding interactions (Fig. 3B). Furthermore, the phosphates of A1 form water-mediated H-bonds with Asp159, while the phosphates of C68 and C69 form water-mediated H-bonds with Glu119 and Ile121. Similar phosphate backbone-mediated interactions with the acceptor stem are also observed in the *Hc*TrpRS·tRNA^Trp^ complex (PDB ID 2DR2). Therefore, it is likely that the overall shape complementarity, rather than the sequence-dependent interactions between the acceptor stem and TrpRS, contributes to the lineage-specific recognition of tRNA^Trp^.

Notably, the base-specific recognition of the major discriminator base G73 was observed in the structure of the *Ec*TrpRS·tRNA^Trp^ complex. The carboxyl group (dual H-bond acceptor) of Glu155 from the AD forms two strong H-bonds with the N1 and N2 atoms (both serve as H-bond donors) of G73 (Fig. 3C). Additionally, Gln152 stacks at one side of G73, facilitating to orient G73 to interact with Glu155. The results from the tRNA-dependent ATP consumption assay revealed that both the Q152A and E155A mutations significantly reduced the catalytic activity of *Ec*TrpRS in comparison to the wild-type enzyme, while the Q152A&E155A double mutation diminished the activity further (Fig. 3D), supporting the critical roles of these two residues in tRNA^Trp^ recognition, as observed in the crystal structure of the *Ec*TrpRS·tRNA^Trp^ complex. Consistently, it has been reported that the enzymatic activity of *Bs*TrpRS in charging *B. subtilis* tRNA^Trp^ decreases by 33% when Glu153 (equivalent to Glu155 in *Ec*TrpRS) is substituted with Asp, which has a shorter side chain, and this activity reduces even more dramatically when Glu153 is mutated to Lys or Gly, both of which lack the H-bond acceptor property [46]. Sequence alignments revealed that both Gln152 and Glu155 in *Ec*TrpRS are strictly conserved across all bacterial TrpRSs, whereas they are replaced by Arg and Pro, respectively, in eukaryotic and archaeal TrpRSs (Supplementary Fig. S2). These substitutions result in the loss of crucial H-bonding interactions with G73 of bacterial tRNA^Trp^, thereby explaining the inefficiency of cross-species charging of bacterial tRNA^Trp^ by *Hc*TrpRS [19]. Interestingly, the significant role of the direct interactions between G73 and the residues Gln152 and Glu155 in the recognition of tRNA^Trp^ is consistent with the observation that the base 73 is one of the “operational RNA code” bases within the tRNA acceptor stem, which determines the specific groove of a tRNA that will be recognized by its AARS [47]. Moreover, both residues Gln152 and Glu155 are situated within the “urzyme” of bacterial TrpRS, the core ancestral peptide of bacterial TrpRS [48], highlighting their key roles in tRNA^Trp^ recognition.

Instead of G73 in bacterial tRNA^Trp^, eukaryotic and archaeal tRNA^Trp^ possess A at position 73 (Fig. 1B) [49]. *In silico* mutation of G73 to A results in the loss of specific H-bonding interactions with Glu155, as both the N1 atom of A73 and the carbonyl oxygen of Glu155 act as H-bond acceptors (Fig. 3E). Furthermore, mutations G73U and G73C may lead to the loss of interactions with Glu155 due to increased distances. Consequently, the aminoacylation efficiency of G73A, G73U, and G73C mutants of *B. subtilis* tRNA^Trp^ by *Bs*TrpRS decreased by ∼9-fold, 17-fold, and 20-fold, respectively, when compared to the wild-type *B. subtilis* tRNA^Trp^. In contrast, the activity of A73G mutant of bovine tRNA^Trp^ with respect to *Bs*TrpRS was approximately three-fold higher than that of the wild-type bovine tRNA^Trp^ [44]. Therefore, by specifically forming H-bonding interactions with a conserved glutamate (Glu155 in *Ec*TrpRS), G73 functions as a positive determinant in the selection of the cognate substrate tRNA^Trp^ for bacterial TrpRS.

The previous studies revealed that the A73 of bovine tRNA^Trp^ was recognized through H-bonding with the main chain of Lys102 and stacking with the side chain of Arg106 in *Hc*TrpRS (Fig. 3C) [20]. Both residues are located on the NTD of *Hc*TrpRS, resulting in the A73 of bovine tRNA^Trp^ being oriented outward to point to the NTD. Thus, A73 is rotated by ∼90° when compared to the orientation of G73 of *Ec*TrpRS-bound *E*.*coli* tRNA^Trp^ (Fig. 3C). The amino acid-accepting CCA end is right behind the discriminator nucleotide 73 in the sequence of tRNA^Trp^, and the dramatically different conformation of nucleotide 73 in bacterial and eukaryotic tRNA^Trp^ may influence the way of the following CCA end approaches the active site of bacterial and eukaryotic TrpRSs. However, the CCA end of *E*.*coli* tRNA^Trp^ cannot be observed in the density map, probably due to that the extension of the 3′ CCA end of *E*.*coli* tRNA^Trp^ into active site cavity requires an “open–closed” conformation of *Ec*TrpRS. Notably, the residues adjacent to the 3′ CCA binding site, as well as the l-Trp binding site, exhibit lower conservation in comparison to those surrounding the ATP binding site between *Ec*TrpRS and *Hc*TrpRS (Fig. 3F), suggesting that the 3′ CCA binding site may serve as a promising target for the development of selective bacterial TrpRS inhibitors as potential antimicrobial agents.

### Hit identification of tirabrutinib and its analogues as *Ec*TrpRS selective inhibitors

**Figure 4.**
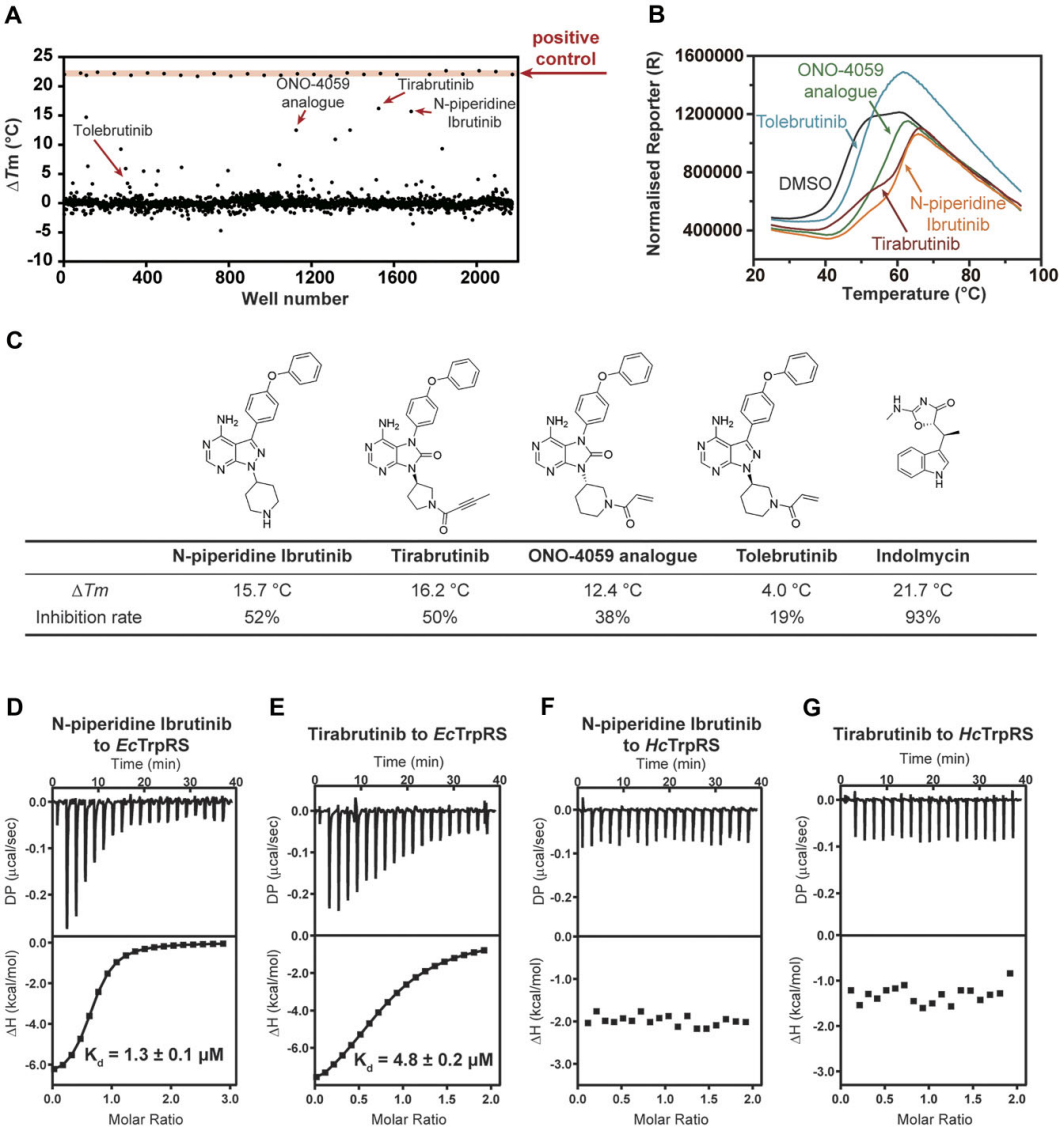
Identification of tirabrutinib and its analogues as selective inhibitors of bacterial TrpRS. (**A**) The scatterplot for the results of compounds screening against *Ec*TrpRS using the fluorescence-based TSA, with indolmycin serving as a positive control. (**B**) The thermal melting profile of *Ec*TrpRS in the presence of tirabrutinib and its analogues. Each curve is an average of three measurements. (**C**) The chemical structures and activities of tirabrutinib and its analogues. (**D, E**) ITC titrations of *N*-piperidine ibrutinib and tirabrutinib to *Ec*TrpRS. (**F, G**) ITC titrations of *N*-piperidine ibrutinib and tirabrutinib to *Hc*TrpRS.

To date, inhibitors targeting bacterial TrpRS, such as indolmycin, chuangxinmycin, and the intermediate product analogue Trp-SA [27, 30, 50], primarily bind to the l-Trp and/or ATP binding sites within the active site cavity, and there have been no published studies regarding inhibitors that target the tRNA binding site of bacterial TrpRS. In this study, a total of 2160 active molecules were screened against *Ec*TrpRS at 100 μM concentration using fluorescence-based protein TSA, with indolmycin serving as the positive control and 1% DMSO as the negative control. Among those, 2144 compounds resulted in analyzable melting curves of *Ec*TrpRS, and their shifts on the melting temperature (*T*_m_) of *Ec*TrpRS were recorded (Fig. 4A). This screening identified 20 compounds that shifted the melting temperature (*T*_m_) of *Ec*TrpRS by greater than **±**4°C (Supplementary Fig. S3). Interestingly, a specific class of Bruton’s tyrosine kinase (BTK) inhibitors with analogous chemical structures was found to both enhance the *T*_m_ values of *Ec*TrpRS and exhibit certain inhibitory activities against *Ec*TrpRS. Among these, *N*-piperidine ibrutinib [51], tirabrutinib, ONO-4059 analogue [52], and tolebrutinib [53] were shown to increase the Δ*T*_m_ of *Ec*TrpRS by 15.7°C, 16.2°C, 12.4°C, and 4.0°C, respectively, at a concentration of 100 μM, and they inhibited the PPi production activity of *Ec*TrpRS with the inhibitory rates of 52%, 50%, 38%, and 19%, respectively, at a concentration of 10 μM (Fig. 4B and C). The structure of these BTK inhibitors can be divided into three constituent parts: the upper portion features two benzene rings connected by an ether bond; the central part is a bicyclic pyrimidine; and the lower portion comprises either a pyrrolidine or piperidine (Fig. 4C). Some other representative BTK inhibitors were also examined, including the approved BTK covalent inhibitors such as ibrutinib [54], IBT6A, IBT4A, and PCI-29732 [55]. However, these inhibitors did not produce significant changes in the *T*_m_ value nor did they exhibit considerable inhibitory rates against *Ec*TrpRS (Supplementary Table S2).

Subsequently, ITC was conducted to quantify the binding affinities of the active compounds. The *K*_d_ value for *N*-piperidine ibrutinib binding to *Ec*TrpRS was determined to be 1.3 ± 0.1 μM, whereas the *K*_d_ value for tirabrutinib to *Ec*TrpRS was found to be 4.8 ± 0.2 μM (Fig. 4D and E). As a control, the binding affinity of indolmycin to *Ec*TrpRS was determined to be 804 ± 54 nM in the absence of ATP and 503 ± 31 nM in the presence of 1 mM ATP (Supplementary Fig. S4A and B). Notably, both tirabrutinib and *N*-piperidine ibrutinib exhibited no binding affinities for *Hc*TrpRS (Fig. 4F and G). While the chemical structures of tirabrutinib and its analogues are dissimilar to indolmycin, chuangxinmycin, and Trp-SA, there is no hit for their binding mode to *Ec*TrpRS.

### Tirabrutinib and analogues represent a class of novel amino acid-tRNA dual-site inhibitors against bacterial TrpRS

**Figure 5.**
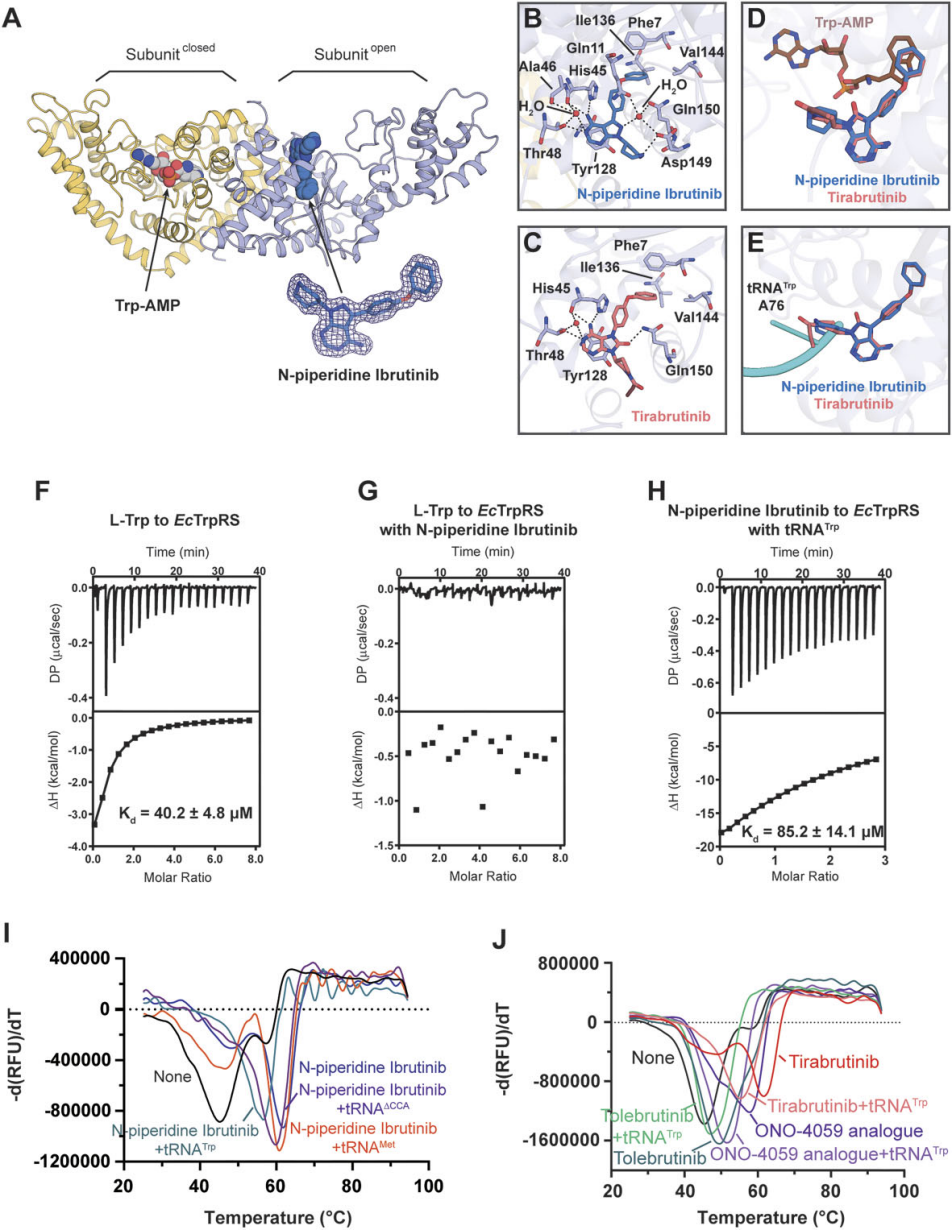
The binding modes of *N*-piperidine ibrutinib and tirabrutinib to *Ec*TrpRS. (**A**) The overall structure of *Ec*TrpRS in complex with *N*-piperidine ibrutinib exhibits an “open–closed” asymmetric conformation, wherein the closed subunit bound with the co-purified intermediate product Trp-AMP and the open subunit bound with *N*-piperidine ibrutinib. The 2*F*_o_–*F*_c_ omit map surrounding *N*-piperidine ibrutinib is shown as mesh and contoured at 1.0*σ*. (**B**) The interactions between *N*-piperidine ibrutinib and *Ec*TrpRS. (**C**) The interactions of tirabrutinib with *Ec*TrpRS. (**D**) Structural superimposition of the compound-bound subunit with the Trp-AMP-bound subunit indicates that the upper benzene ring of the compounds occupies the l-Trp binding site within the active site cavity. (**E**) Structural superimposition of the compound-bound *Ec*TrpRS with the tRNA^Trp^-bound *Hc*TrpRS (PDB ID 2DR2) reveals that the pyrrolidine or piperidine moiety of the compounds interferes with the A76 of tRNA^Trp^. (**F**) The ITC titration of l-Trp to *Ec*TrpRS. (**G**) The ITC titration of l-Trp to *Ec*TrpRS in the presence of 200 μM of *N*-piperidine ibrutinib. (**H**) The titration curve of *N*-piperidine ibrutinib to *Ec*TrpRS in the presence of 100 μM of tRNA^Trp^. (**I**) Thermal melting profiles of *Ec*TrpRS in the presence of *N*-piperidine ibrutinib as well as its combinations with tRNA^Trp^, tRNA^Trp^(ΔCCA), or tRNA^Met^. (**J**) Thermal melting profiles of *Ec*TrpRS in the presence of tirabrutinib, its analogues, and their combinations with tRNA^Trp^. Each curve represents the average of three measurements.

To enhance the understanding of the interactions between these compounds and bacterial TrpRS, *Ec*TrpRS was co-crystallized with *N*-piperidine ibrutinib and tirabrutinib, and the complex structures were resolved to 1.6 and 2.0 Å, respectively (Supplementary Table S1). Similar to the tRNA^Trp^-free structure of *Ec*TrpRS we previously solved, *Ec*TrpRS exhibits an “open–closed” asymmetric conformation in both of these structures. The closed subunit is bound with a molecule of intermediate product Trp-AMP that is synthesized in *E*.*coli* cells and co-purified with the *Ec*TrpRS protein, while the open subunit is engaged with a molecule of either *N*-piperidine ibrutinib or tirabrutinib that were supplemented during crystal growth process (Fig. 5A and Supplementary Fig. S6A). Both *N*-piperidine ibrutinib and tirabrutinib bind with similar conformation within the active site cavity on the AD domain (Fig. 5B and C). For *N*-piperidine ibrutinib, the upper benzene ring is situated within a hydrophobic pocket formed by Phe7, Ile136, and Val144, while the other benzene ring engages in π–π stacking interaction with His45. The amino group on the central pyrimidine ring forms two H-bonds with His45, while the adjacent pyrimidine nitrogen atom forms another H-bond with the side chain of Thr48. Additionally, the adjacent pyrimidine nitrogen atom interacts with a water molecule coordinated by His45, Ala46, and Thr48. The pyrimidine ring engages in π–π stacking interaction with Tyr128. Furthermore, the nitrogen atom on the pyrazole ring forms a water-mediated H-bond with Gln11, Asp149, and Gln150. The nitrogen atom in the piperidine ring H-bonds with Asp149 (Fig. 5B). The interactions involving the two benzene rings and the pyrimidine ring of *N*-piperidine ibrutinib are preserved in the corresponding structures of tirabrutinib. However, instead of the water-mediated H-bond of the pyrazole ring nitrogen, the carbonyl group within the five-membered ring of tirabrutinib directly H-bonds with Gln150 (Fig. 5C). Unlike the piperidine ring of *N*-piperidine ibrutinib, the but-2-ynamide group in tirabrutinib extends toward the solvent region without establishing any specific interactions with *Ec*TrpRS.

The structural comparison between Trp-AMP-bound subunit and the compound-bound subunit revealed that the upper benzene ring of *N*-piperidine ibrutinib and tirabrutinib occupies the l-Trp binding site of *Ec*TrpRS without interfering with the ATP binding site (Fig. 5D). Notably, superposition with the structure of the *Hc*TrpRS·tRNA^Trp^ complex (PDB ID 2DR2) indicated that the pyrrolidine and piperidine moieties may interfere with the approach of the 3′ end A76 of tRNA^Trp^ to the active site to accept the l-Trp moiety (Fig. 5E). The affinity for l-Trp binding to *Ec*TrpRS was determined to be 40.2 ± 4.8 μM using ITC. In contrast, no binding of l-Trp to *Ec*TrpRS could be detected in the presence of 200 μM *N*-piperidine ibrutinib, indicating competition between l-Trp and *N*-piperidine ibrutinib for *Ec*TrpRS binding (Fig. 5F and G). In a similar manner, we evaluated the binding affinity of *N*-piperidine ibrutinib to *Ec*TrpRS using ITC in the presence of 100 μM tRNA^Trp^. The *K*_d_ value was determined to be 85.2 ± 14.1 μM (Fig. 5H), which decreased by ∼65-fold in comparison to the affinity observed in the absence of tRNA^Trp^ (*K*_d_ = 1.3 ± 0.1 μM) (Fig. 4D). While the binding of tRNA^Trp^ to *Ec*TrpRS did not yield stable thermal signaling, we also investigated the potential competition between *N*-piperidine ibrutinib and tRNA^Trp^ using TSA. The introduction of 200 μM tRNA^Trp^ resulted in a reduction of the Δ*T*_m_ value of *Ec*TrpRS bound to *N*-piperidine ibrutinib by 5.1°C (Fig. 5I). In contrast, as a control, tRNA^Met^ induced only a marginal Δ*T*_m_ reduction of 1.0°C at the same concentration. Importantly, after removing the 3′-CCA end of tRNA^Trp^, the tRNA^Trp^(ΔC74C75A76) variant at 200 μM concentration could only reduce the Δ*T*_m_ of *N*-piperidine ibrutinib by 2.1°C, supporting that it is the 3′-CCA end of tRNA^Trp^ directly competes with *N*-piperidine ibrutinib (Fig. 5I).

Furthermore, the Δ*T*_m_ values of *Ec*TrpRS with tirabrutinib, ONO-4059 analogue, and tolebrutinib were all reduced following the addition of tRNA^Trp^, suggesting that tRNA^Trp^ interfered with the binding of these compounds to *Ec*TrpRS (Fig. 5J). Notably, control experiments confirmed that neither tRNA^Trp^ nor the compounds alone produced detectable fluorescence signals in TSA in the absence of *Ec*TrpRS (Supplementary Fig. S5).

Collectively, the structural and biophysical analyses indicated that these compounds may function as potential dual-site inhibitors by simultaneously occupying both the l-Trp and the tRNA^Trp^ binding sites within the active site cavity of bacterial TrpRS, thereby providing valuable insights for future drug design.

Notably, the l-Trp and tRNA 3′ CCA end binding sites are less conserved between bacterial and human TrpRSs when compared to their ATP binding sites (Fig. 3F). The residues Phe7, Ile136, and Val144 that build a hydrophobic pocket in *Ec*TrpRS are replaced by Tyr159, Ser288, and Ile307, respectively, in *Hc*TrpRS. Thus, when the compounds were docked into the active site cavity of *Hc*TrpRS, the upper benzene ring of the compounds exhibited a reduced hydrophobic interaction with *Hc*TrpRS when compared to *Ec*TrpRS (Supplementary Fig. S6B). Moreover, His45 is conserved in bacterial TrpRS, while a glutamate is present at the corresponding position in *Hc*TrpRS (Supplementary Fig. S2). The stacking interaction between His45 and the second benzene ring of the compounds is critical; however, this interaction is completely absent in *Hc*TrpRS. The residue Thr48 in *Ec*TrpRS corresponds to Leu202 in *Hc*TrpRS, which loses its H-bonding interaction with the pyrimidine nitrogen atom of *N*-piperidine ibrutinib and tirabrutinib. Additionally, the bulky residues Trp203 and Phe267 in *Hc*TrpRS may create spatial conflicts with the compounds. This observation may explain the lack of affinity between the compounds and *Hc*TrpRS from a structural perspective (Supplementary Fig. S6B).

## Discussion

In the co-crystal structure of the *Ec*TrpRS·tRNA^Trp^ complex, *Ec*TrpRS adopts an “open–open” conformation to simultaneously capture two *E*.*coli* tRNA^Trp^ molecules in a symmetric manner. This “open–open” conformation of *Ec*TrpRS provides partial support for our previous hypothesis that at least one subunit of the homodimeric bacterial TrpRS must adopt an open conformation to enable the functional binding of tRNA^Trp^ [17]. However, the formation of the intermediate product Trp-AMP necessitates that at least one subunit of bacterial TrpRS transitions to a closed conformation (PDB ID 8I1W) [17]; therefore, this “open–open” conformation does not accurately reflect the aminoacylation state of *Ec*TrpRS, which may partially explain why the CCA end of tRNA^Trp^ has not been stabilized within the active site cavity of *Ec*TrpRS in the structure of the *Ec*TrpRS·tRNA^Trp^ complex. It is probable that, at the aminoacylation state, there is only one tRNA^Trp^ molecule binding to the asymmetric “open–closed” *Ec*TrpRS, with the 3′ CCA end inserting into the active site cavity of the closed subunit while the anticodon triplet interacts with the CTD of the open subunit.

In pursuit of capturing the aminoacylation state complex, the “open–closed” *Ec*TrpRS was used in the crystallization assay. However, *E. coli* tRNA^Trp^ can react with and hydrolyze the Trp-AMP bound within the closed subunit of *Ec*TrpRS, thereby facilitating the transition from the closed conformation to the open conformation. We then generated the *E*.*coli* tRNA^Trp^ variants with truncations at A76, C75A76, and C74C75A76. Among those, tRNA^Trp^ (ΔA76) has been successfully co-crystallized with *Ec*TrpRS. Unexpectedly, in the absence of A76, which is the terminal nucleotide of tRNA^Trp^ responsible for attacking the intermediate product Trp-AMP and accepting the l-Trp moiety, two tRNA^Trp^(ΔA76) molecules were captured by *Ec*TrpRS in a symmetric “open–open” conformation. We hypothesize that the high concentration of tRNA^Trp^(ΔA76) in the crystallization drops promotes the opening of *Ec*TrpRS, resulting in the release of Trp-AMP to the solvent and the subsequent hydrolysis. Consequently, the “open–closed” *Ec*TrpRS transitioned to the “open–open” conformation, and was then co-crystallized with two molecules of tRNA^Trp^(ΔA76). Interestingly, tRNA^Trp^(ΔA76) remained to consume ATP, despite a reduced reaction rate compared to the full-length tRNA^Trp^ (Supplementary Fig. S7). The ATP consumption by *Ec*TrpRS in the presence of tRNA^Trp^(ΔC75A76) and tRNA^Trp^(ΔC74C75A76) was significantly reduced. Probably, these double or triple nucleotide truncated variants of tRNA^Trp^ may facilitate the capture of the crystal structure of the *Ec*TrpRS·tRNA^Trp^ complex at the aminoacylation conformation. It is noteworthy that the crystal packing force may also promote the formation of unexpected complex structures, particularly in the case of bacterial TrpRS, which demonstrates significant conformational changes upon interaction with different ligands. For example, when the crystals of *Bacillus stearothermophilus* TrpRS, co-crystallized with the low-molecular-weight product Trp-3′ATP, were transferred from the mother liquid to ammonium sulfate for data collection, the *B. stearothermophilus* TrpRS underwent a transition to an intermediate Trp-AMP bound complex, which was accompanied by a change in the unit cell [11, 56]. Therefore, it may be necessary to explore different growth conditions to successfully capture the desired asymmetric *Ec*TrpRS·tRNA^Trp^ complex in crystals.

The recognition of tyrosine and tryptophan by TyrRS and TrpRS occurs late in the evolution of the genetic code, and bacterial and eukaryotic TrpRSs have evolved distinct mechanisms in the recognition of l-Trp [18]. Structure-based alignments indicate that the ATP binding site exhibits a higher degree of conservation between eukaryotic and bacterial TrpRSs, whereas the 3′ CCA binding site of tRNA^Trp^ as well as the l-Trp binding site has lower conservation, highlighting the great potential of these two sites in the development of bacterial-selective TrpRS inhibitors as potential antimicrobial agents. While indolmycin and chuangxinmycin are predominantly the l-Trp binding site inhibitors of bacterial TrpRS, our study identifies a class of BTK inhibitors that selectively inhibit bacterial TrpRS by simultaneously occupying both the l-Trp site and tRNA site. This amino acid-tRNA dual-site inhibitory mechanism was first characterized in halofuginone, a herbal-derived inhibitor of eukaryotic prolyl-tRNA synthetases (ProRSs) [57, 58]. The structure-guided design of halofuginone derivatives targeting bacterial ProRS and threonyl-tRNA synthetase (ThrRS) has yielded amino acid-tRNA dual-site inhibitors with promising antibacterial activities [59–61]. While ProRS and ThrRS both belong to class II AARSs, the first inhibitor that was identified to utilize the tRNA 3′ CCA end binding site of class I AARSs is reveromycin A, a natural product inhibitor of eukaryotic IleRS [62, 63]. As reveromycin A is a sole tRNA site inhibitor, to the best of our knowledge, no amino acid-tRNA dual-site inhibitors for TrpRS and other class I AARSs have been reported. Therefore, our study not only offers the first structural insights into the mechanism of the long-discussed lineage-specific recognition of tRNA^Trp^ by bacterial TrpRS, but also provides a new direction for the future development of TrpRS-directed antibacterial agents.

## Supplementary Material

gkaf466_Supplemental_File

## Data Availability

Atomic coordinates and structure factors for the reported crystal structures have been deposited with the Protein Data Bank under accession codes 9LPC (*Ec*TrpRS·tRNA^Trp^), 9LOT (*Ec*TrpRS·TrpAMP·N-piperidine ibrutinib), and 9LPD (*Ec*TrpRS·TrpAMP·tirabrutinib).

## References

[1] Ibba M, Söll D Quality control mechanisms during translation. Science. 1999; 286:1893–7. 10.1126/science.286.5446.1893. 10583945

[2] Ribas de Pouplana L, Schimmel P Aminoacyl-tRNA synthetases: potential markers of genetic code development. Trends Biochem Sci. 2001; 26:591–6. 10.1016/S0968-0004(01)01932-6. 11590011

[3] Ibba M, Söll D Aminoacyl-tRNA synthesis. Annu Rev Biochem. 2000; 69:617–50. 10.1146/annurev.biochem.69.1.617. 10966471

[4] Ahn YH, Oh SC, Zhou S et al. Tryptophanyl-tRNA synthetase as a potential therapeutic target. Int J Mol Sci. 2021; 22:4523 10.3390/ijms22094523. 33926067 PMC8123658

[5] Moen SO, Edwards TE, Dranow DM et al. Ligand co-crystallization of aminoacyl-tRNA synthetases from infectious disease organisms. Sci Rep. 2017; 7:223 10.1038/s41598-017-00367-6. 28303005 PMC5428304

[6] Zhou M, Dong X, Shen N et al. Crystal structures of *Saccharomyces cerevisiae* tryptophanyl-tRNA synthetase: new insights into the mechanism of tryptophan activation and implications for anti-fungal drug design. Nucleic Acids Res. 2010; 38:3399–413. 10.1093/nar/gkp1254. 20123733 PMC2879500

[7] Laowanapiban P, Kapustina M, Vonrhein C et al. Independent saturation of three TrpRS subsites generates a partially assembled state similar to those observed in molecular simulations. Proc Natl Acad Sci USA. 2009; 106:1790–5. 10.1073/pnas.0812752106. 19174517 PMC2644116

[8] Retailleau P, Weinreb V, Hu M et al. Crystal structure of tryptophanyl-tRNA synthetase complexed with adenosine-5' tetraphosphate: evidence for distributed use of catalytic binding energy in amino acid activation by class I aminoacyl-tRNA synthetases. J Mol Biol. 2007; 369:108–28. 10.1016/j.jmb.2007.01.091. 17428498 PMC2715954

[9] Retailleau P, Huang X, Yin Y et al. Interconversion of ATP binding and conformational free energies by tryptophanyl-tRNA synthetase: structures of ATP bound to open and closed, pre-transition-state conformations. J Mol Biol. 2003; 325:39–63. 10.1016/S0022-2836(02)01156-7. 12473451

[10] Ilyin VA, Temple B, Hu M et al. 2.9 A crystal structure of ligand-free tryptophanyl-tRNA synthetase: domain movements fragment the adenine nucleotide binding site. Protein Sci. 2000; 9:218–31. 10.1110/ps.9.2.218. 10716174 PMC2144547

[11] Doublié S, Bricogne G, Gilmore C et al. Tryptophanyl-tRNA synthetase crystal structure reveals an unexpected homology to tyrosyl-tRNA synthetase. Structure. 1995; 3:17–31. 10.1016/S0969-2126(01)00132-0. 7743129

[12] Antonellis A, Green ED The role of aminoacyl-tRNA synthetases in genetic diseases. Annu Rev Genomics Hum Genet. 2008; 9:87–107. 10.1146/annurev.genom.9.081307.164204. 18767960

[13] Guth E, Farris M, Bovee M et al. Asymmetric amino acid activation by class II histidyl-tRNA synthetase from *Escherichia coli*. J Biol Chem. 2009; 284:20753–62. 10.1074/jbc.M109.021311. 19487703 PMC2743188

[14] Shen N, Zhou M, Yang B et al. Catalytic mechanism of the tryptophan activation reaction revealed by crystal structures of human tryptophanyl-tRNA synthetase in different enzymatic states. Nucleic Acids Res. 2008; 36:1288–99. 10.1093/nar/gkm1153. 18180246 PMC2275098

[15] Hughes SJ, Tanner JA, Hindley AD et al. Functional asymmetry in the lysyl-tRNA synthetase explored by molecular dynamics, free energy calculations and experiment. BMC Struct Biol. 2003; 3:5 10.1186/1472-6807-3-5. 12787471 PMC165585

[16] Ward WH, Fersht AR Asymmetry of tyrosyl-tRNA synthetase in solution. Biochemistry. 1988; 27:1041–9. 10.1021/bi00403a029. 3365365

[17] Xiang M, Xia K, Chen B et al. An asymmetric structure of bacterial TrpRS supports the half-of-the-sites catalytic mechanism and facilitates antimicrobial screening. Nucleic Acids Res. 2023; 51:4637–49. 10.1093/nar/gkad278. 37070195 PMC10201369

[18] Yang XL, Otero FJ, Skene RJ et al. Crystal structures that suggest late development of genetic code components for differentiating aromatic side chains. Proc Natl Acad Sci USA. 2003; 100:15376–80. 10.1073/pnas.2136794100. 14671330 PMC307575

[19] Xu F, Chen X, Xin L et al. Species-specific differences in the operational RNA code for aminoacylation of tRNA^Trp^. Nucleic Acids Res. 2001; 29:4125–33. 10.1093/nar/29.20.4125. 11600701 PMC60218

[20] Shen N, Guo L, Yang B et al. Structure of human tryptophanyl-tRNA synthetase in complex with tRNA reveals the molecular basis of tRNA recognition and specificity. Nucleic Acids Res. 2006; 34:3246–58. 10.1093/nar/gkl441. 16798914 PMC1538984

[21] Pang L, Weeks SD, Van Aerschot A Aminoacyl-tRNA synthetases as valuable targets for antimicrobial drug discovery. Int J Mol Sci. 2021; 22:1750 10.3390/ijms22041750. 33578647 PMC7916415

[22] Kwon NH, Fox PL, Kim S Aminoacyl-tRNA synthetases as therapeutic targets. Nat Rev Drug Discov. 2019; 18:629–50. 10.1038/s41573-019-0026-3. 31073243

[23] Vondenhoff GHM, Van Aerschot A Aminoacyl-tRNA synthetase inhibitors as potential antibiotics. Eur J Med Chem. 2011; 46:5227–36. 10.1016/j.ejmech.2011.08.049. 21968372

[24] Schimmel P, Tao J, Hill J Aminoacyl tRNA synthetases as targets for new anti-infectives. FASEB J. 1998; 12:1599–609. 10.1096/fasebj.12.15.1599. 9837850

[25] Silvian LF, Wang J, Steitz TA Insights into editing from an Ile-tRNA synthetase structure with tRNA^Ile^ and mupirocin. Science. 1999; 285:1074–7. 10.1126/science.285.5430.1074. 10446055

[26] Sutherland R, Boon RJ, Griffin KE et al. Antibacterial activity of mupirocin (pseudomonic acid), a new antibiotic for topical use. Antimicrob Agents Chemother. 1985; 27:495–8. 10.1128/AAC.27.4.495. 3923922 PMC180082

[27] Williams TL, Yin YW, Carter CW Jr Selective inhibition of bacterial tryptophanyl-tRNA synthetases by indolmycin is mechanism-based. J Biol Chem. 2016; 291:255–65. 10.1074/jbc.M115.690321. 26555258 PMC4697160

[28] Fan S, Lv G, Feng X et al. Structural insights into the specific interaction between *Geobacillus stearothermophilus* tryptophanyl-tRNA synthetase and antimicrobial chuangxinmycin. J Biol Chem. 2022; 298:101580 10.1016/j.jbc.2022.101580. 35031320 PMC8814664

[29] Sun L, Zhang S, Kou S et al. Design, synthesis, and antibacterial activity of derivatives of tryptophanyl-tRNA synthetase inhibitor indolmycin. Eur J Med Chem. 2022; 241:114647 10.1016/j.ejmech.2022.114647. 35963132

[30] Sun L, Zhang S, Hu X et al. Synthesis, resolution, derivatization and antibacterial activity of chuangxinmycin. Future Med Chem. 2019; 11:2877–90. 10.4155/fmc-2019-0209. 31533475

[31] Kao C, Zheng M, Rüdisser S A simple and efficient method to reduce nontemplated nucleotide addition at the 3 terminus of RNAs transcribed by T7 RNA polymerase. RNA. 1999; 5:1268–72. 10.1017/S1355838299991033. 10496227 PMC1369849

[32] Kabsch W XDS. Acta Crystallogr D Biol Crystallogr. 2010; 66:125–32. 10.1107/S0907444909047337. 20124692 PMC2815665

[33] Evans PR, Murshudov GN How good are my data and what is the resolution?. Acta Crystallogr D Biol Crystallogr. 2013; 69:1204–14. 10.1107/S0907444913000061. 23793146 PMC3689523

[34] Vagin A, Teplyakov A Molecular replacement with MOLREP. Acta Crystallogr D Biol Crystallogr. 2010; 66:22–5. 10.1107/S0907444909042589. 20057045

[35] Emsley P, Lohkamp B, Scott WG et al. Features and development of Coot. Acta Crystallogr D Biol Crystallogr. 2010; 66:486–501. 10.1107/S0907444910007493. 20383002 PMC2852313

[36] Murshudov GN, Skubák P, Lebedev AA et al. REFMAC5 for the refinement of macromolecular crystal structures. Acta Crystallogr D Biol Crystallogr. 2011; 67:355–67. 10.1107/S0907444911001314. 21460454 PMC3069751

[37] Williams CJ, Headd JJ, Moriarty NW et al. MolProbity: more and better reference data for improved all-atom structure validation. Protein Sci. 2018; 27:293–315. 10.1002/pro.3330. 29067766 PMC5734394

[38] Chen B, Yi F, Luo Z et al. The mechanism of discriminative aminoacylation by isoleucyl-tRNA synthetase based on wobble nucleotide recognition. Nat Commun. 2024; 15:10817 10.1038/s41467-024-55183-0. 39738040 PMC11685878

[39] Lu F, Xia K, Su J et al. Biochemical and structural characterization of chlorhexidine as an ATP-assisted inhibitor against type 1 methionyl-tRNA synthetase from Gram-positive bacteria. Eur J Med Chem. 2024; 268:116303 10.1016/j.ejmech.2024.116303. 38458107

[40] Niesen FH, Berglund H, Vedadi M The use of differential scanning fluorimetry to detect ligand interactions that promote protein stability. Nat Protoc. 2007; 2:2212–21. 10.1038/nprot.2007.321. 17853878

[41] Yang Y, Xu Y, Yue Y et al. Investigate natural product indolmycin and the synthetically improved analogue toward antimycobacterial agents. ACS Chem Biol. 2022; 17:39–53. 10.1021/acschembio.1c00394. 34908399

[42] Kisselev LL The role of the anticodon in recognition of tRNA by aminoacyl-tRNA synthetases. Prog Nucleic Acid Res Mol Biol. 1985; 32:237–66. 3911276 10.1016/s0079-6603(08)60350-5

[43] Himeno H, Hasegawa T, Asahara H et al. Identity determinants of *E. coli* tryptophan tRNA. Nucleic Acids Res. 1991; 19:6379–82. 10.1093/nar/19.23.6379. 1721699 PMC329181

[44] Guo Q, Gong Q, Tong KL et al. Recognition by tryptophanyl-tRNA synthetases of discriminator base on tRNA from three biological domains. J Biol Chem. 2002; 277:14343–9. 10.1074/jbc.M111745200. 11834741

[45] Xu F, Jiang G, Li W et al. Three G.C base pairs required for the efficient aminoacylation of tRNATrp by tryptophanyl-tRNA synthetase from *Bacillus subtilis*. Biochemistry. 2002; 41:8087–92. 10.1021/bi015881g. 12069601

[46] Jia J, Chen XL, Guo LT et al. Residues Lys-149 and Glu-153 switch the aminoacylation of tRNA(Trp) in *Bacillus subtilis*. J Biol Chem. 2004; 279:41960–5. 10.1074/jbc.M401937200. 15280378

[47] Carter CW Jr., Wills PR Hierarchical groove discrimination by Class I and II aminoacyl-tRNA synthetases reveals a palimpsest of the operational RNA code in the tRNA acceptor-stem bases. Nucleic Acids Res. 2018; 46:9667–83. 10.1093/nar/gky600. 30016476 PMC6182185

[48] Li L, Francklyn C, Carter CW Jr Aminoacylating urzymes challenge the RNA world hypothesis. J Biol Chem. 2013; 288:26856–63. 10.1074/jbc.M113.496125. 23867455 PMC3772232

[49] Sprinzl M, Horn C, Brown M et al. Compilation of tRNA sequences and sequences of tRNA genes. Nucleic Acids Res. 1998; 26:148–53. 10.1093/nar/26.1.148. 9399820 PMC147216

[50] Van de Vijver P, Ostrowski T, Sproat B et al. Aminoacyl-tRNA synthetase inhibitors as potent and synergistic immunosuppressants. J Med Chem. 2008; 51:3020–9. 10.1021/jm8000746. 18438987

[51] Buhimschi AD, Armstrong HA, Toure M et al. Targeting the C481S ibrutinib-resistance mutation in Bruton’s tyrosine kinase using PROTAC-mediated degradation. Biochemistry. 2018; 57:3564–75. 10.1021/acs.biochem.8b00391. 29851337

[52] Yasuhiro T, Yoshizawa T, Birkett JT. et al. ONO-4059, a novel Bruton’s tyrosine kinase (Btk) inhibitor: synergistic activity in combination with chemotherapy in a ABC-DLBCL cell line. Blood. 2013; 122:5151 10.1182/blood.V122.21.5151.5151.

[53] Owens TD, Smith PF, Redfern A et al. Phase 1 clinical trial evaluating safety, exposure and pharmacodynamics of BTK inhibitor tolebrutinib (PRN2246, SAR442168). Clin Transl Sci. 2022; 15:442–50. 10.1111/cts.13162. 34724345 PMC8841436

[54] Byrd JC, Brown JR, O’Brien S et al. Ibrutinib versus ofatumumab in previously treated chronic lymphoid leukemia. N Engl J Med. 2014; 371:213–23. 10.1056/NEJMoa1400376. 24881631 PMC4134521

[55] Honigberg LA, Smith AM, Sirisawad M et al. The Bruton tyrosine kinase inhibitor PCI-32765 blocks B-cell activation and is efficacious in models of autoimmune disease and B-cell malignancy. Proc Natl Acad Sci USA. 2010; 107:13075–80. 10.1073/pnas.1004594107. 20615965 PMC2919935

[56] Carter CW Jr., Coleman DE Crystallization of substrate and product analog complexes of tryptophanyl-tRNA synthetase. Fed Proc. 1984; 43:2981–3. 6500072

[57] Zhou H, Sun L, Yang XL et al. ATP-directed capture of bioactive herbal-based medicine on human tRNA synthetase. Nature. 2013; 494:121–4. 10.1038/nature11774. 23263184 PMC3569068

[58] Jain V, Yogavel M, Kikuchi H et al. Targeting prolyl-tRNA synthetase to accelerate drug discovery against malaria, leishmaniasis, toxoplasmosis, cryptosporidiosis, and coccidiosis. Structure. 2017; 25:1495–1505. 10.1016/j.str.2017.07.015. 28867614

[59] Guo J, Chen B, Yu Y et al. Discovery of novel tRNA-amino acid dual-site inhibitors against threonyl-tRNA synthetase by fragment-based target hopping. Eur J Med Chem. 2020; 187:111941 10.1016/j.ejmech.2019.111941. 31821989

[60] Guo J, Chen B, Yu Y et al. Structure-guided optimization and mechanistic study of a class of quinazolinone-threonine hybrids as antibacterial ThrRS inhibitors. Eur J Med Chem. 2020; 207:112848 10.1016/j.ejmech.2020.112848. 32980741

[61] Cheng B, Cai Z, Luo Z et al. Structure-guided design of halofuginone derivatives as ATP-aided inhibitors against bacterial prolyl-tRNA synthetase. J Med Chem. 2022; 65:15840–55. 10.1021/acs.jmedchem.2c01496. 36394909

[62] Miyamoto Y, Machida K, Mizunuma M et al. Identification of *Saccharomyces cerevisiae* isoleucyl-tRNA synthetase as a target of the G1-specific inhibitor reveromycin A. J Biol Chem. 2002; 277:28810–4. 10.1074/jbc.M203827200. 12050165

[63] Chen B, Luo S, Zhang S et al. Inhibitory mechanism of reveromycin A at the tRNA binding site of a class I synthetase. Nat Commun. 2021; 12:1616 10.1038/s41467-021-21902-0. 33712620 PMC7955072

