# Supplementary material for: The mechanism of lineage-specific tRNA recognition by bacterial tryptophanyl-tRNA synthetase and its implications for inhibitor discovery
Source: Nucleic Acids Res. 2025 Jun 4;53(10):gkaf466. doi: 10.1093/nar/gkaf466 (PMC12135181; doi:10.1093/nar/gkaf466)
Supplement: gkaf466_Supplemental_File [file gkaf466_supplemental_file.pdf]

Supplementary Information

The mechanism of lineage-specific tRNA recognition by bacterial tryptophanyl-tRNA synthetase and its implications for inhibitor discovery

Xiaoying Peng[1,2,#], Kaijiang Xia[1,2,#], Lingzhen Xiao[1,2], Haoran Qi[1,2], Qingting Huang[1,2], Manli Xiang[1,2], Lu Han[1,2], Haipeng Qiu[1,2], Qiong Gu[1], Bingyi Chen[1,2,*], Huihao Zhou[1,2,*]

[1]State Key Laboratory of Anti-Infective Drug Discovery and Development, School of Pharmaceutical Sciences, Sun Yat-sen University, Guangzhou 510006, China.

[2]Guangdong Provincial Key Laboratory of Chiral Molecule and Drug Discovery, School of Pharmaceutical Sciences, Sun Yat-sen University, Guangzhou 510006, China.

[#]These authors contributed equally to this work.

* To whom correspondence should be addressed. Tel: +86 20 39943350; Email: zhuihao@mail.sysu.edu.cn

Correspondence may also be addressed to Bingyi Chen. E-mail: chenby86@mail.sysu.edu.cn

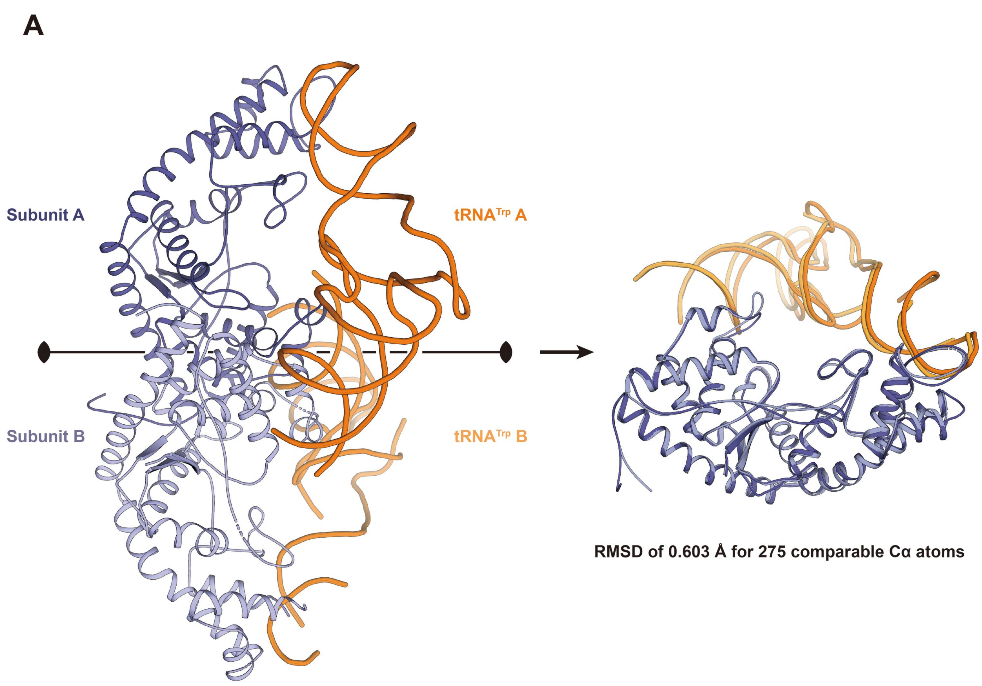

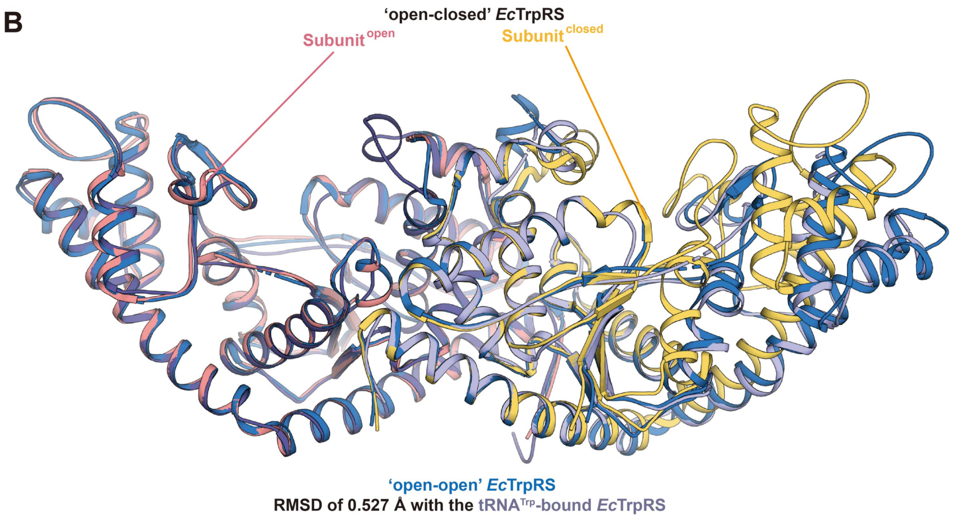

Supplementary Figure S1. The *Ec*TrpRS·tRNA^{Trp} complex adopts a symmetric 'open-open' conformation. **(A)** Structural superimposition of the two subunits of the *Ec*TrpRS dimer exhibits nearly perfect alignments for their structures and their associated tRNA^{Trp} molecules. **(B)** A structural comparison of tRNA^{Trp}-bound *Ec*TrpRS (light purple) with the tRNA^{Trp}-free *Ec*TrpRS at both the 'open-open' conformation (PDB ID 5V0I, blue) and the 'open-closed' asymmetric conformation (PDB ID 8I1W, subunit^{open} in pink and subunit^{closed} in yellow) revealed that tRNA^{Trp}-bound *Ec*TrpRS adopts the 'open-open' conformation. The overall RMSD is 0.527 Å for 529 comparable Cα atoms between tRNA^{Trp}-bound *Ec*TrpRS and the *Ec*TrpRS at the 'open-open' conformation.

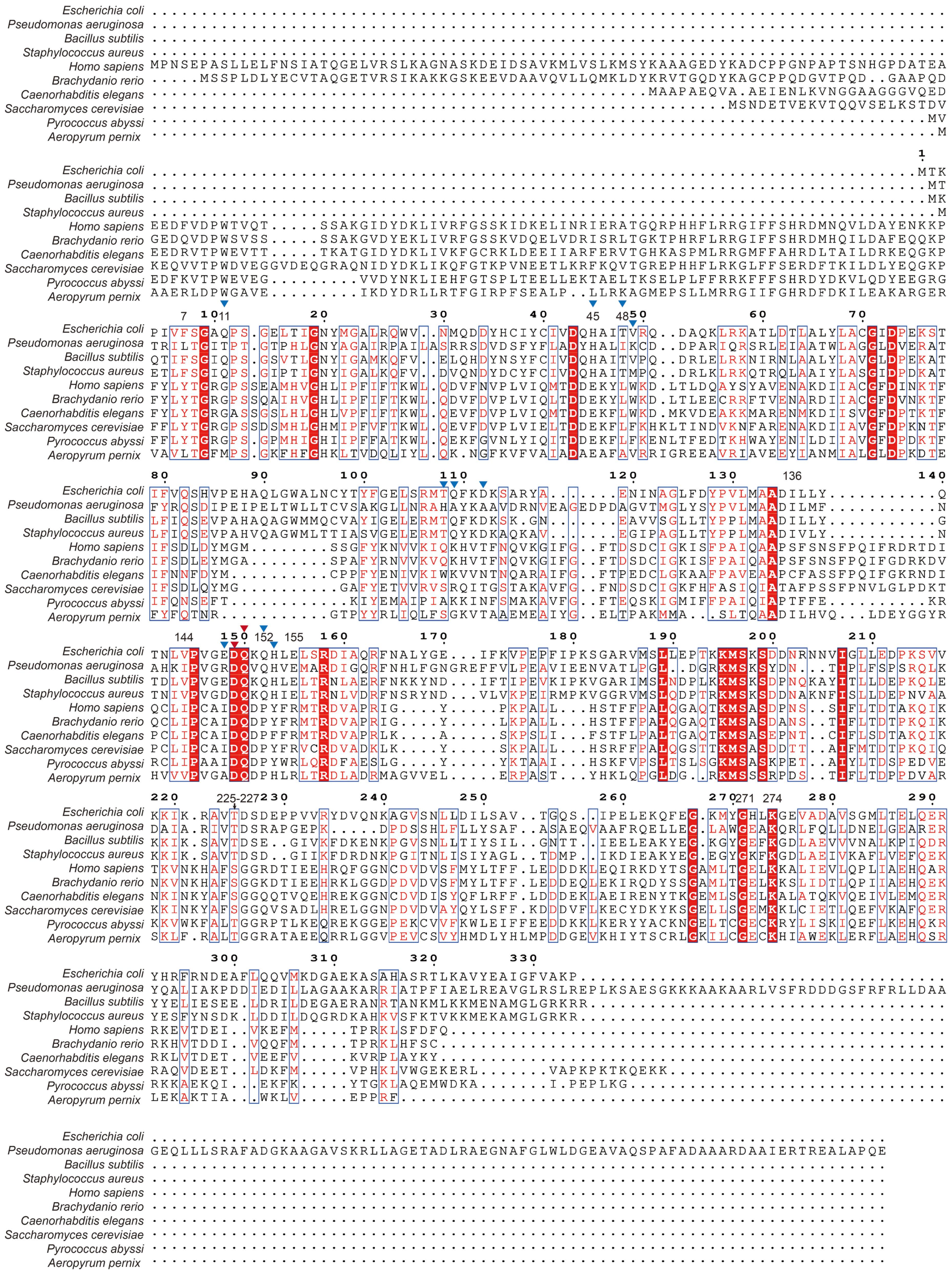

Supplementary Figure S2. Structure-based sequence alignments of *Ec*TrpRS with representative TrpRSs from various species. Residues that may interact with the 3′ CCA of *E.coli* tRNA^{Trp} are indicated by triangles, with blue triangles representing non-conserved residues between *Ec*TrpRS and *Hc*TrpRS, and red triangles denoting conserved residues.

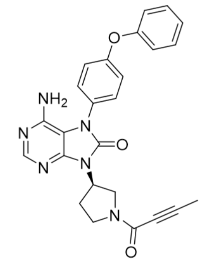

tirabrutinib
16.2 °C

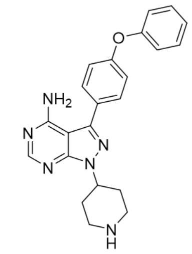

N-piperidine ibrutinib
15.7 °C

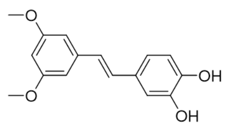

3'-Hydroxypterostilbene
14.7 °C

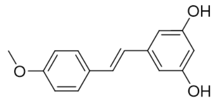

4'-Methoxyresverstrol
12.5 °C

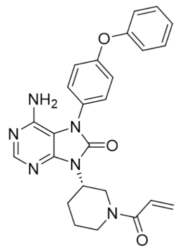

ONO-4059 analogue
12.4 °C

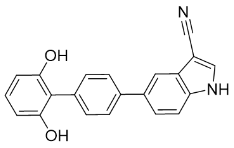

MT 63-78
10.9 °C

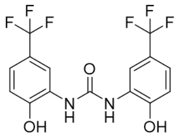

NS 1643
9.3 °C

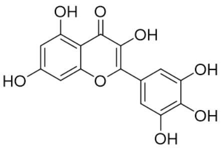

Myricetin
9.3 °C

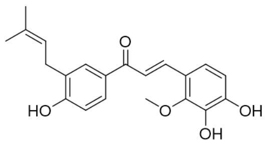

Licochalcone D
6.6 °C

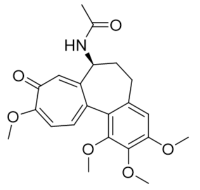

3-Demethylcolchicine
6.3 °C

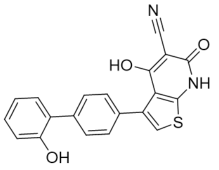

A-769662
6.1 °C

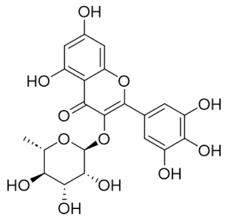

Myricitrin
5.9 °C

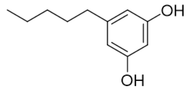

Olivetol
5.6 °C

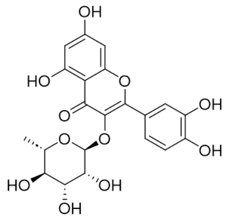

Quercitrin
5.5 °C

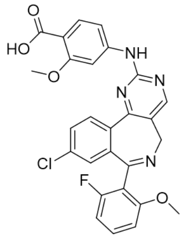

Alisertib
5.5 °C

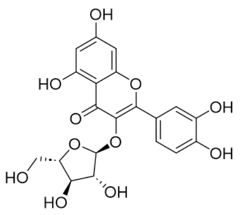

Avicularin
4.7 °C

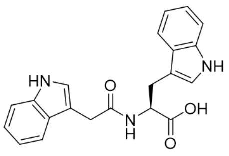

Indole-3-acetyl-L-tryptophan
4.6 °C

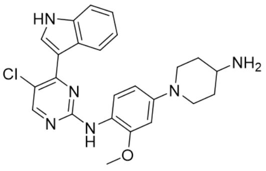

AZD-3463
4.1 °C

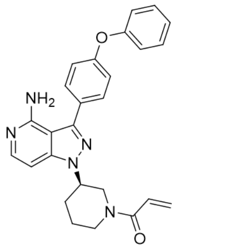

Tolebrutinib
4.0 °C

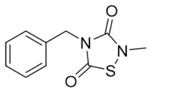

TDZD-8
-4.7 °C

Supplementary Figure S3. The chemical structures of compounds that were identified to bind to *Ec*TrpRS using fluorescence-based thermal shift assay. A total of twenty compounds were found to shift the melting temperature (T_m) of *Ec*TrpRS by greater than ± 4 °C.

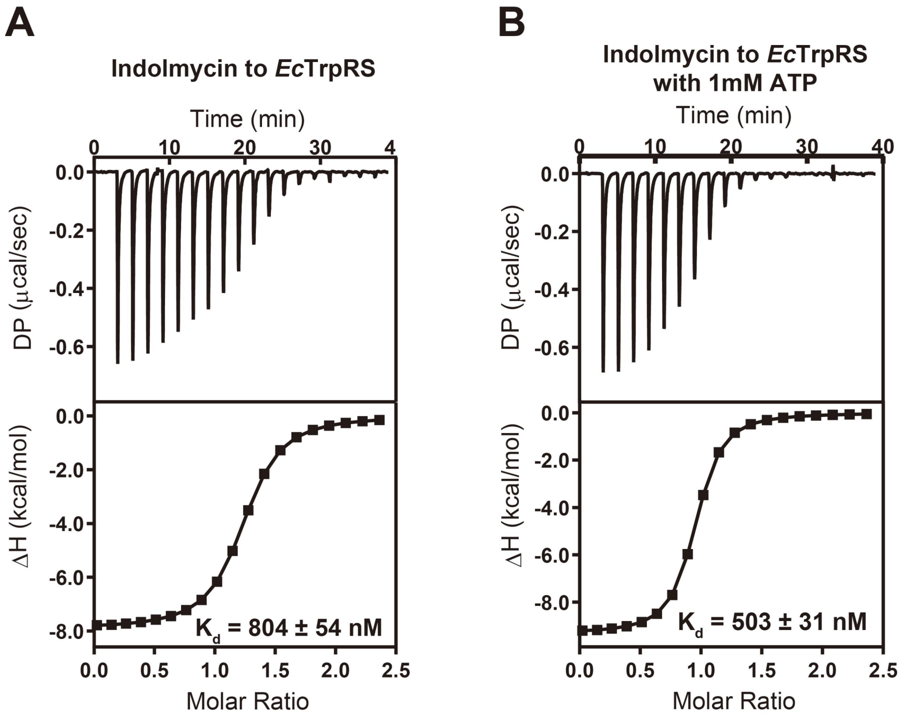

Supplementary Figure S4. The affinity of indolmycin binding to *Ec*TrpRS was measured using ITC. **(A)** The ITC titration of indolmycin to *Ec*TrpRS. **(B)** The ITC titration of indolmycin to *Ec*TrpRS in the presence of ATP.

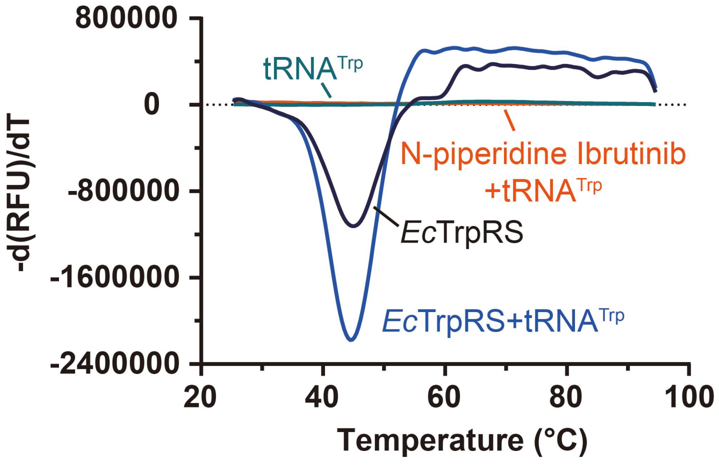

Supplementary Figure S5. The thermal melting profile of tRNA^{Trp}, *Ec*TrpRS and N-piperidine ibrutinib. It was observed that tRNA^{Trp} did not exhibit a significant fluorescence signal; therefore, it is unlikely to interfere with the interpretation of the thermal denaturation process of *Ec*TrpRS.

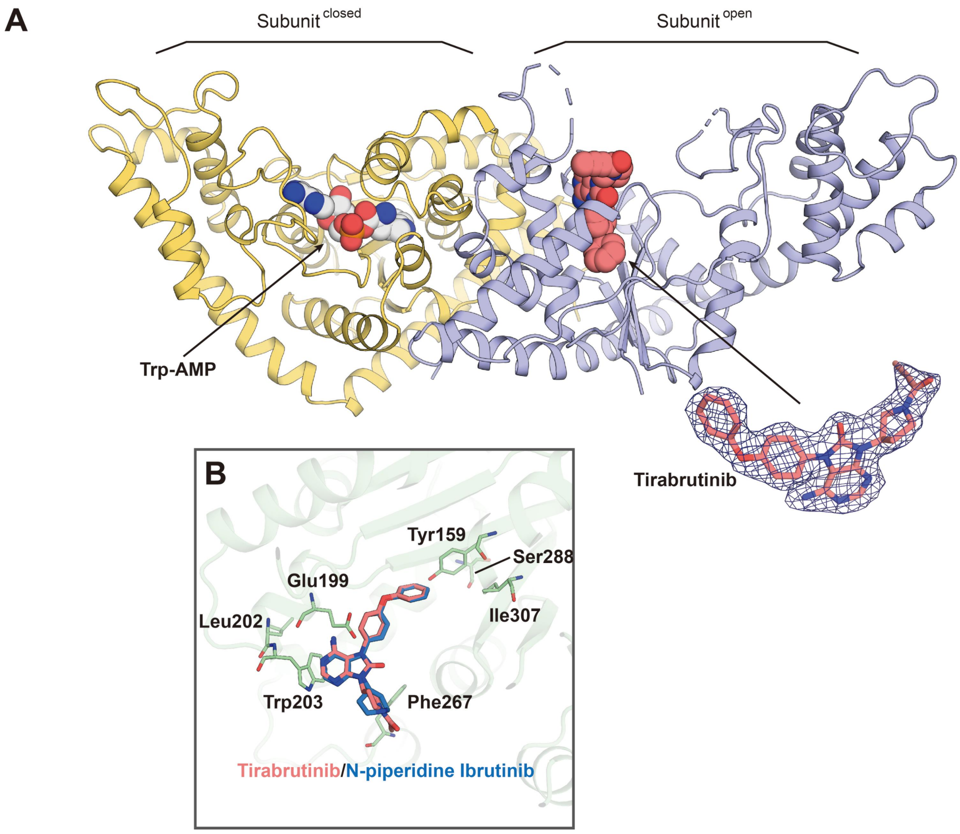

Supplementary Figure S6. The binding of compounds to TrpRS. **(A)** The overall structure of *Ec*TrpRS in complex with tirabrutinib. One subunit of *Ec*TrpRS binds with the intermediate product Trp-AMP, while the other subunit binds with tirabrutinib. *Ec*TrpRS shows an 'open-closed' asymmetric conformation. The $2F_o$-F_c omit map surrounding tirabrutinib is shown as blue mesh and contoured at 1.0 σ. **(B)** The docking of N-piperidine ibrutinib and tirabrutinib to *Hc*TrpRS revealed that these compounds lack hydrophobic interactions and most hydrogen bonding interactions. Furthermore, these compounds may cause spatial conflicts with Trp203 and Phe267 of *Hc*TrpRS.

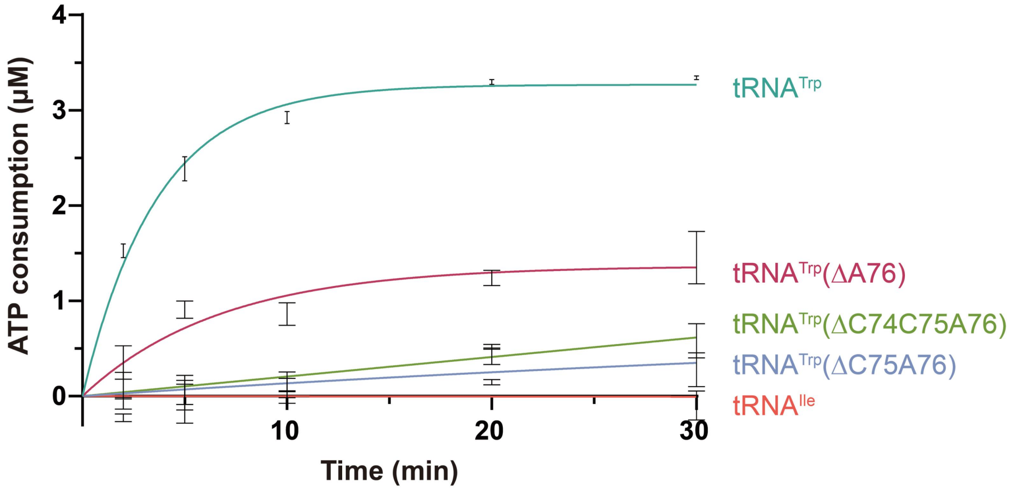

Supplementary Figure S7. The aminoacylation activity of TrpRS against in vitro transcribed $tRNA^{Trp}$ as well as its variants with truncations at A76, C75A76, and C74C75A76. The $tRNA^{Trp}$(ΔA76) remained to consume ATP, albeit at a reduced rate compared to the wild-type $tRNA^{Trp}$. Data are presented as means ± SD (n = 3 independent experiments).

Supplementary Table

Table S1. Data collection and refinement statistics.

	*Ec*TrpRS·tRNA^{Trp}	*Ec*TrpRS·Trp-AMP·N-piperidine ibrutinib	*Ec*TrpRS·Trp-AMP·tirabrutinib
**PDB code**	9LPC	9LOT	9LPD
**Data collection**			
Wavelength (Å)	0.9796	0.9792	0.9785
Resolution (Å)	89.51-2.82(2.87-2.82)	75.41-1.59(1.67-1.59)	76.32-2.05(2.16-2.05)
Space group	$P1$	$P2_1$	$P2_1$
Cell dimensions			
a, b, c (Å)	60.79, 63.21, 91.63	61.77, 79.65, 78.36	61.77, 79.43, 79.28
α, β, γ (°)	98.50, 97.25, 100.04	90.00, 105.78, 90.00	90.00, 105.71, 90.00
Unique reflections	30805(1346)	98739(14351)	46067(6713)
R_{merge}[b]	0.041(0.491)	0.094(1.535)	0.077(0.473)
Average $I/\sigma(I)$	21.2(1.3)	9.0(1.4)	8.4(2.4)
Completeness (%)	97.35(83.34)	99.9(99.6)	98.6(99.2)
Redundancy	3.3(2.8)	5.6(4.1)	3.2(3.3)
**Refinement**			
Resolution (Å)	59.12-2.82	54.49-1.59	30.65-2.05
No. reflections	30742	92139	45967
R_{work}[c] /R_{free}[d] (%)	0.236/0.271	0.190/0.205	0.233/0.250
No. non-hydrogen atoms			
Protein	4570	4959	4977
RNA	2879		

Ligand/ion		92	97
Water oxygen atoms	7	470	180
Mean B factor (Å^2)	94.0	22.8	47.0
RMSD bond (Å)	0.010	0.006	0.011
RMSD angle (°)	1.207	1.286	1.253
Ramachandran plot (%)			
Favored	93.03	96.28	96.59
Allowed	6.97	3.72	3.41
Outliers	0.00	0.00	0.00

[a]Values in parentheses are for the highest resolution shell.

[b]$R_{merge} = \sum_h \sum_l |I(h)_l - \langle I(h) \rangle| / \sum_h \sum_l I(h)_l$, where $I(h)_l$ is the lth observation of the reflection h and $\langle I(h) \rangle$ is the weighted average intensity for all observations l of reflection h.

[c]$R_{work} = \sum h ||F_{obs}(h)| - |F_{cal}(h)|| / \sum_h |F_{obs}(h)|$, where $F_{obs}(h)$ and $F_{cal}(h)$ are the observed and calculated structure factors for reflection h respectively.

[d]R_{free} was calculated as R_{work} using 5% of the reflections which were selected randomly and omitted from refinement.

Table S2. The *ΔT*m values and inhibitory rates of the compounds against *Ec*TrpRS.

Compd.	Name	Structure	ΔT_m (°C) [a]	Inhibitory rate (%) [b]
1	N-piperidine Ibrutinib	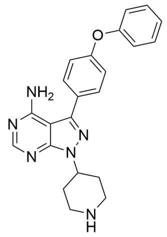	15.7	52
2	Tirabrutinib	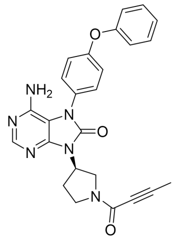	16.2	50
3	ONO-4059 analogue	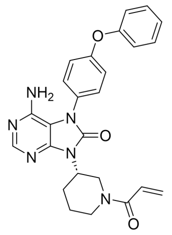	12.4	38
4	Tolebrutinib	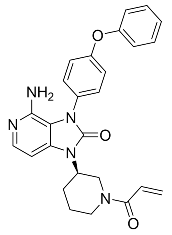	4.0	19
5	Ibrutinib	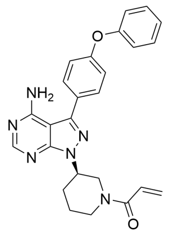	< 2	12
6	IBT6A	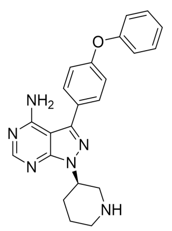	< 2	9

7	PCI-29732	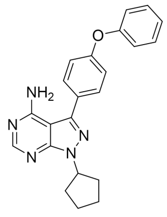	< 2	< 5
8	IBT4A	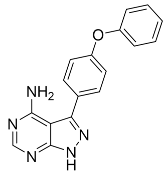	< 2	< 5
9	Indolmycin	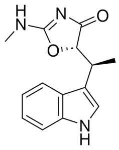	22.5	93

[a]ΔT_m is the difference between the T_m values of *Ec*TrpRS with and without 0.1 mM compound.

[b]The inhibition rate (%) represents the ratio of the decrease in *Ec*TrpRS activity in the presence of 10 μM of each compound. The ΔT_m values and inhibitory rates are the average of three independent experiments.

Table S3. Primers for tRNA^{Trp} transcription in vitro.

Primer name	Sequence (5' - 3')
tRNA^{Trp}	
Primer1-1	**TAATACGACTCACTATA**AGGGGCGTAGTTCAATTGGTAGAGCACCGGTCTCCAAAACC
Primer1-2	TGGCAGGGGCGGAGAGACTCGAACTCCCAACACCCGGTTTTGGAGACCGGTGCT
Primer1-3	**TAATACGACTCACTATA**AGGGGCGTAG
Primer1-4	*UG*GCAGGGGCGGAGAGACTCGA
$\text{tRNA}^{\text{Trp}}(\Delta\text{A76})$	
Primer2-2	GGCAGGGGCGGAGAGACTCGAACTCCCAACACCCGGTTTTGGAGACCGGTGCT
Primer2-4	*GG*CAGGGGCGGAGAGACTCGA
$\text{tRNA}^{\text{Trp}}(\Delta\text{C75A76})$	
Primer3-2	GCAGGGGCGGAGAGACTCGAACTCCCAACACCCGGTTTTGGAGACCGGTGCT
Primer3-4	*GC*AGGGGCGGAGAGACTCGA
$\text{tRNA}^{\text{Trp}}(\Delta\text{C74C75A76})$	
Primer4-2	CAGGGGCGGAGAGACTCGAACTCCCAACACCCGGTTTTGGAGACCGGTGCT
Primer4-4	*CA*GGGGCGGAGAGACTCGA

Primer2-1 and Primer2-3 for $\text{tRNA}^{\text{Trp}}(\Delta\text{A76})$, Primer3-1 and Primer3-3 for $\text{tRNA}^{\text{Trp}}(\Delta\text{C75A76})$, and Primer4-1 and Primer4-3 for $\text{tRNA}^{\text{Trp}}(\Delta\text{C74C75A76})$ are identical to Primer1-1 and Primer1-3.
